# Simpson's Paradox in COVID-19 Case Fatality Rates: A Mediation Analysis of Age-Related Causal Effects

**DOI:** 10.1109/TAI.2021.3073088

**Published:** 2021-04-14

**Authors:** Julius von Kügelgen, Luigi Gresele, Bernhard Schölkopf

**Affiliations:** Max Planck Institute for Intelligent Systems 28325 72076 Tübingen Germany; University of Cambridge 2152 Cambridge CB2 1PZ U.K.; Max Planck Institute for Intelligent Systems 28325 72076 Tübingen Germany

**Keywords:** Causal inference, COVID-19, mediation analysis, Simpson's paradox

## Abstract

We point out an instantiation of Simpson's paradox in COVID-19 case fatality rates (cfrs): comparing a large-scale study from China (February 17) with early reports from Italy (March 9), we find that cfrs are lower in Italy for every age group, but higher overall. This phenomenon is explained by a stark difference in case demographic between the two countries. Using this as a motivating example, we introduce basic concepts from mediation analysis and show how these can be used to quantify different direct and indirect effects when assuming a coarse-grained causal graph involving country, age, and case fatality. We curate an age-stratified cfr dataset with $>$750 k cases and conduct a case study, investigating total, direct, and indirect (age-mediated) causal effects between different countries and at different points in time. This allows us to separate age-related effects from others unrelated to age and facilitates a more transparent comparison of cfrs across countries at different stages of the COVID-19 pandemic. Using longitudinal data from Italy, we discover a sign reversal of the direct causal effect in mid-March, which temporally aligns with the reported collapse of the healthcare system in parts of the country. Moreover, we find that direct and indirect effects across 132 pairs of countries are only weakly correlated, suggesting that a country's policy and case demographic may be largely unrelated. We point out limitations and extensions for future work, and finally, discuss the role of causal reasoning in the broader context of using AI to combat the COVID-19 pandemic.

*Impact Statement*—During a global pandemic, understanding the causal effects of risk factors such as age on COVID-19 fatality is an important scientific question. Since randomised controlled trials are typically infeasible or unethical in this context, causal investigations based on observational data—such as the one carried out in this article—will, therefore, be crucial in guiding our understanding of the available data. Causal inference, in particular mediation analysis, can be used to resolve apparent statistical paradoxes; help educate the public and decision-makers alike; avoid unsound comparisons; and answer a range of causal questions pertaining to the pandemic, subject to transparently stated assumptions. Our exposition helps clarify how mediation analysis can be used to investigate direct and indirect effects along different causal paths and thus serves as a stepping stone for future studies of other important risk factors for COVID-19 besides age.

## I. Introduction

The 2019–20 coronavirus pandemic originates from the SARS-CoV-2 virus, which causes the associated infectious respiratory disease COVID-19. After an outbreak was identified in Wuhan, China, in December 2019, cases started being reported across multiple countries all over the world, ultimately leading to the World Health Organization declaring it a pandemic on March 11, 2020 [1]. As of September 28, 2020, the pandemic led to more than 33 million confirmed cases and one million fatalities across 188 countries [2]. One of the most cited indicators regarding COVID-19 is the reported *case fatality rate* (cfr), which indicates the proportion of confirmed cases, which end fatally. In addition to the *total* cfr, cfrs are often also reported separately *by age* since cfrs differ significantly across different age groups, with older people statistically at higher risk.

In this article, we show how tools from causal inference and, in particular, mediation analysis can be used to interpret COVID-19 case fatality data. We motivate our investigation by pointing out what could be a textbook example of Simpson's paradox in comparing cfrs between China and Italy, suggesting opposite conclusions depending on whether the data are analyzed in aggregate or age-stratified form, as shown in Section II. This example illustrates how a traditional statistical analysis provides insufficient understanding of the data, and thus needs to be augmented by additional assumptions about the underlying causal relationships. In Section III, we therefore postulate a coarse-grained causal model for comparing age-specific COVID-19 cfr data across different countries. We then review different types of (direct and indirect) causal effects, and motivate them in the context of our assumed model as different questions about COVID-19 case fatality in Section IV.

As one of our contributions, we curated a dataset involving 756004 confirmed COVID-19 cases and 68508 fatalities, separated into age groups of ten-year intervals (0–9, 10–19, etc.), reported from 11 different countries from Africa, Asia, Europe, and South America and the Diamond Princess cruise ship, which, together with an interactive notebook containing all our analyses, is publicly available. We use this dataset, in combination with the proposed coarse-grained model, to perform a case study, as shown in Section V. Tracing the evolution of direct and indirect age-mediated effects of country (China or Italy) on case fatality from early March to late May 2020 allows to discover trends that may otherwise remain hidden in the data, e.g., a reversal in the sign of the direct effect in mid-March that temporally aligns with a reported “collapse” of the healthcare system in parts of Italy [3]. Moreover, we compute direct and indirect effects for 132 pairs of countries and, thus, identify countries whose total cfrs are particularly adversely affected by their case demographic. We further find that indirect (age-related) effects are strongly correlated with a country's population's median age, but only weakly with direct effects.

Due to the limited availability of age-stratified fatality data, our model is relatively simple, and we do not claim novelty in the causal methodology. However, this article constitutes, to the best of our knowledge, the first application of causal analysis to better understand the role of mediators, such as age in the context of COVID-19. While the use of cfr data may be problematic due to selection bias from differences in testing (which we discuss in VI), we emphasize that our causal framework may likewise be applied to more comprehensive datasets once available. We thus hope that this article can serve as a stepping stone for further studies to gain better insight into the mechanisms underlying COVID-19 fatality using a principled and transparent causal framework.

## II. Simpson's Paradox in Comparing cfrs Between China and Italy

**Fig. 1.**
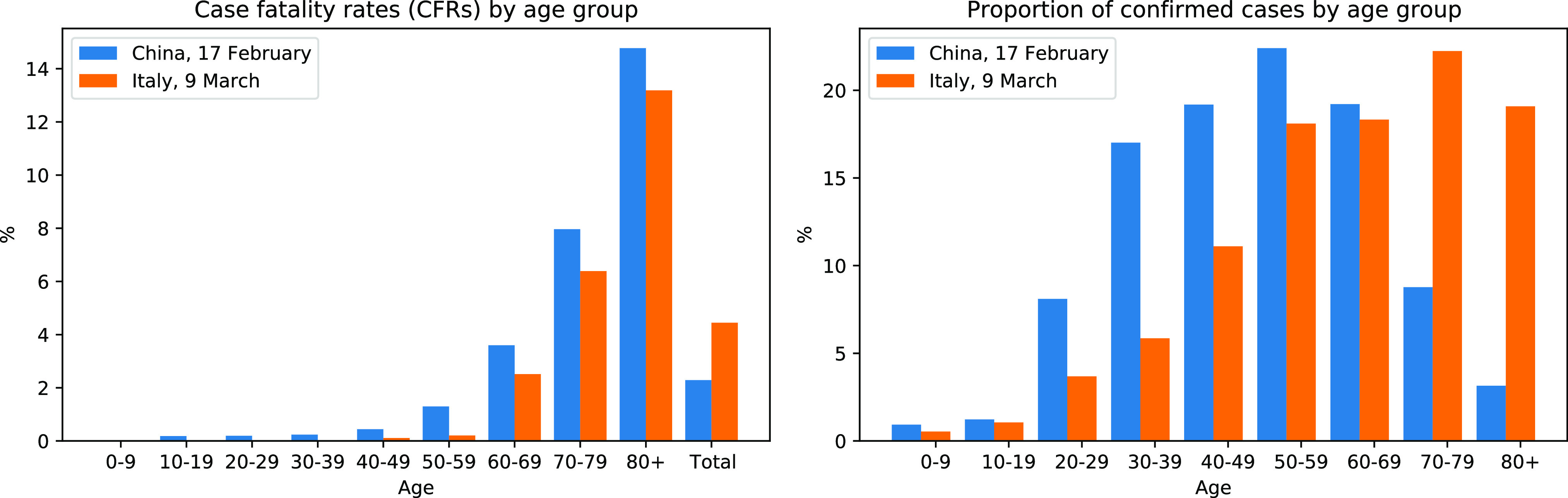
(Left) COVID-19 cfrs in Italy and China by age group and in aggregated form (“Total”), i.e., including all *confirmed* cases and fatalities up to the time of reporting (see legend). (Right) Proportion of cases within each age group.

When comparing COVID-19 cfrs for different age groups (i.e., the proportion of confirmed COVID-19 cases within a given age group that end fatal) reported by the Chinese Center for Disease Control and Prevention [4] with preliminary cfrs from Italy, as reported on March 9 by the Italian National Institute of Health [5], a surprising pattern can be observed: *for all age groups,* cfr*s in Italy are lower than those in China, but the total* cfr* in Italy is higher than that in China.* This is illustrated in Fig. 1—see Appendix VIII-D for exact numbers. It constitutes a textbook example of a statistical phenomenon known as *Simpson's paradox* (or *reversal*), which refers to the observation that aggregating data across subpopulations (here, age groups) may yield opposite trends (and, thus, lead to reversed conclusions) from considering subpopulations separately [6].

*How can such a pattern be explained?* The key to understanding the phenomenon lies in the fact that we are dealing with *relative* frequencies: the cfrs shown in percent in Fig. 1 (left) are ratios and correspond to the conditional probabilities of fatality given a case from a particular age group and country. However, such percentages conceal the absolute numbers of cases within each age group. Considering these absolute numbers sheds light on how the phenomenon can arise: the distribution of cases across age groups differs significantly between the two countries, i.e., there is a statistical association between the country of reporting and the case demographic. In particular, Italy recorded a much higher proportion of confirmed cases in older patients, as illustrated in Fig. 1 (right).

While most cases in China fell into the age range of 30–59, the majority of cases reported in Italy were in people aged 60 and over who are generally at higher risk of dying from COVID-19, as illustrated by the increase in cfrs with age for both countries. The observed difference may partly stem from the fact that the Italian population in general is older than the Chinese one with median ages of 45.4 and 38.4, respectively, but additional factors, such as different testing strategies and patterns, in the social contacts among older and younger generations, e.g., [7]–[9], may also play a role. In summary, the larger share of confirmed cases among elderly people in Italy, combined with the fact that the elderly are generally at higher risk when contracting COVID-19 explains the mismatch between total and age-stratified cfrs and, thus, gives rise to Simpson's paradox in the data.

We note that other instances of Simpson's paradox have already been observed in the context of epidemiological studies. When recording tuberculosis deaths in New York City and Richmond, Virginia, in 1910, for example, it was noted that even though overall tuberculosis mortality was lower in New York than in Richmond, the opposite was true when populations where stratified according to ethnicity [10].

## III. Causal Model for COVID-19 cfr Data

While the previous reasoning provides a perfectly consistent explanation in a *statistical* sense, the phenomenon may still seem puzzling as it defies our *causal* intuition—similar to how an optical illusion defies our visual intuition. Humans appear to naturally extrapolate conditional probabilities to read them as causal effects, which can lead to inconsistent conclusions and may leave one wondering: *how can the disease in Italy be less fatal for the young, less fatal for the old, but more fatal for the people overall?* It is for this reason of ascribing causal meaning to probabilistic statements that the reversal of (conditional) probabilities in II is perceived as and referred to as a “paradox” [11]–[13].

The aspiration to extract causal conclusions from data is particularly strong during a pandemic, when many inherently causal questions are naturally asked. For example, politicians and citizens may want to evaluate different strategies to fight the disease by asking interventional or counterfactual (*“what would have happened if...?”*) questions. However, it is a well-known scientific mantra that *correlation does not imply causation*, and observational data (like that in Fig. 1) alone are generally insufficient to draw causal conclusions. While correlations can be seen as a result of underlying causal mechanisms [14], different causal models can explain the same statistical association patterns equally well [15]. Additional assumptions on the underlying causal structure are therefore necessary to guide reasoning based on observational data.

### A. Included Variables

We consider the following three variables for comparing COVID-19 cfrs across different countries.

1) The *country* $C$ in which a confirmed case is *reported*, modeled as a categorical variable.
2) The *age group* $A$ of a positively tested patient, an ordinal variable with ten-year intervals as values.
3) The *medical outcome, or fatality,* $F$, a binary variable indicating whether a patient has deceased by the time of reporting ($F=1$) or not ($F=0$).

### B. Data Generating Process and Causal Graph

**Fig. 2.**
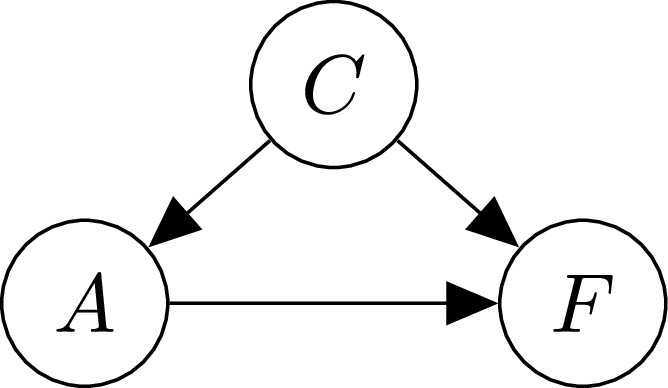
Assumed causal graph: within this view age, $A$ acts as a *mediator* of the effect of country $C$ on case fatality $F$.

We assume the causal graph shown in Fig. 2, motivated by thinking of the following data-generating process.

1) choosing a country $C$ at random;
2) given the selected country $C$, sampling a positively tested patient with age group $A$;
3) conditional on the choice of $C$ and $A$, sampling the case fatality $F$.

This is clearly a very simple and coarse-grained view of what is known to be a complex underlying phenomenon. As a consequence, we abstract away various influences and mechanisms within the arrows in Fig. 2.

1) $(C\rightarrow A)$ captures that the case demographic is country-dependent. This difference might be due to a general difference in age demographic between countries, but other mechanisms, such as inter-generational mixing or age-targeted social distancing, may also play a role.
2) $(A\rightarrow F)$ encodes that COVID-19 is more dangerous for the elderly: age seems to have a causal effect on fatality.
3) $(C\rightarrow F)$ summarizes country-specific influences on case fatality other than age, e.g., medical infrastructure, such as availability of hospital beds and ventilators, local expertise and pandemic-preparedness (e.g., from experience with SARS), air pollution levels, and other nonpharmaceutical interventions and policies which may indirectly affect case fatality via caseload, influencing the capacity of the healthcare system. We will refer to the combination of all these effects as a country's *approach*.

We emphasize that we do *not* explicitly model the infection process, but consider only drivers of fatality conditional on having tested positive, see VI for further discussion.

A similar causal model to that in Fig. 2 (see [16]) was subsequently used to assay another instance of Simpson's paradox in COVID-19 cfr data: in that case, ethnicity rather than country of origin takes the role of a common cause of age group and fatality, and age that of a mediator [17].^1^^1^The overall cfr for “White, Non-Hispanic” people in the US was higher than for other ethnic groups, but, when stratifying by age, the cfr for “White, Non-Hispanic” was lower in almost all age groups (except 0–4 year olds). As in our example, this reversal can be explained by a difference in case demographics across different ethnic groups.

### C. Observational Sample and Causal Sufficiency

We assume that cfrs and case demographic are based on an observational sample and, thus, constitute estimates of $P(F=1|A=a, C=c)$ and $P(A=a|C=c)$, respectively. In addition, we assume causal sufficiency, meaning that all common causes of $C, A,$ and $ F$ are observed (i.e., there are no hidden confounders). While this is a strong assumption, it is necessary to reason about causal effects and also perhaps not entirely unrealistic in our setting: all unobserved variables described earlier can be seen as latent mediators.

## IV. Total, Direct, and Indirect (Age-Mediated) Causal Effects on Case Fatality

Having clearly stated our assumptions, we can now answer causal queries within the model postulated in III. In this section, we review definitions of different causal effects (following the treatment of Pearl [18]) and provide interpretations thereof by phrasing them as questions about different aspects of the cfr data in Fig. 1. We defer a discussion of issues, such as identifiability, under different conditions to Appendix VIII-A. Example calculations for each defined quantity using the data from Fig. 1 can be found in Appendix VIII-B. Throughout, we denote an intervention that externally fixes a variable $X$ to a particular value $x$ (as opposed to conditioning on it) using the notation $do(X=x)$ [15].

### A. Total Causal Effect (tce)

First, we may ask about the overall causal effect of the choice of country on case fatality.

$Q_\textsc {tce}$: *“What would be the effect on fatality of changing country from China to Italy?”*

The answer is called the average tce.

**Definition 1**

**(****tce****)** The tce of a binary treatment $T$ on $Y$ is defined as the interventional contrast

\begin{align*}
\textsc {tce}_{0\rightarrow 1} =& \mathbb {E}_{Y|do(T=1)}[Y|do(T=1)] \\
&- \mathbb {E}_{Y|do(T=0)}[Y|do(T=0)]. \tag{1}
\end{align*}

In our setting (i.e., according to the causal graph in Fig. 2), the country $C$ takes the role of a treatment that affects the medical outcome $F$ (denoted by $T$ and $Y$ in Definition 1, respectively), and (subject to causal sufficiency) the tce is simply given by the difference in total cfrs.

### B. “Why?”: Beyond Total Effects via Mediation Analysis

While computing the tce is the principled way to quantify the *total* causal influence, it does not help us understand what drives a difference between two countries, i.e., *why* it exists in the first place: we may also be interested in the *mechanisms* that give rise to different cfrs observed across countries. Since the age of patients was crucial for explaining the instance of Simpson's paradox in II, we now seek to better understand the role of age as a mediator of the effect of country on fatality. This seems particularly relevant from the perspective of countries, which—unable to influence the age distribution of the general population—only have limited control over the case demographic and, thus, may wish to factor out age-related effects. However, such potential mediators are not reflected within the tce, as evident from the absence of the age variable $A$ from (1).

The country $C$ causally influences fatality $F$ along two different paths: a direct path $C\rightarrow F$, giving rise to a *direct effect*;^2^^2^Recall that the direct effect of country on case fatality is likely mediated by additional variables, which are subsumed in $C\rightarrow F$ in the current view—see VI for further discussion. and an indirect path $C\rightarrow A \rightarrow F$ mediated by $A$, giving rise to an *indirect effect*. The tce of $C$ on $F$ thus comprises both direct and indirect effects. Quantifying such direct and indirect effects is referred to as *mediation analysis* [18]. The main challenge is that any changes to the country $C$ propagate along both direct and indirect paths, making it difficult to isolate the different effects. The key idea is therefore to let changes propagate only along one path while controlling or fixing the effect along the other.

### C. Controlled Direct Effect (cde)

The simplest way to measure a direct effect is by changing the treatment (country) while keeping the mediator fixed at a particular value. For example, we may ask about the causal effect for a particular age group, such as 50–59 year olds.

$Q_{\textsc {cde}(50-59)}$: *“For 50–59 year olds, is it safer to get the disease in China or in Italy?”*

Because it involves actively *controlling* the value of the mediator, the answer to such a query is referred to as the average cde. It is defined as follows.

**Definition 2**

**(****cde****)** The cde of a binary treatment $T$ on an outcome $Y$ with mediator $X=x$ is

\begin{align*}
\textsc {cde}_{0\rightarrow 1}(x) =& \mathbb {E}[Y|do(T=1, X=x)] \\
&- \mathbb {E}[Y|do(T=0, X=x)]. \tag{2}
\end{align*}

For our assumed setting, the cde is given by the difference of cfrs for a given age group. A practical shortcoming of the cde is that it is often difficult or even impossible to control both the treatment and the mediator.^3^^3^In medical settings, for example, one generally cannot easily control individual downstream effects of a drug within the body, such as fixing, e.g., blood glucose levels while changing treatments. Another problem is that the cde does not provide a global quantity for comparing baseline and treatment: in our setting, there is a different cde *for each age group*. However, we may instead want to measure a direct effect at the *population level*.

### D. Natural Direct Effect (nde)

Instead of fixing the mediator to a specific value (selecting a particular age group), we can consider the hypothetical question of what would happen under a change in treatment (country) if the mediator (age) kept behaving as it would under the control, i.e., as if the change only propagated along the direct path. This corresponds to asking about the effect of switching country without affecting the age distribution across confirmed cases.

$Q_\text{NDE}$: *“For the Chinese case demographic, would the Italian approach have been better?”*

As it relies on the mediator (age) distribution under the control (China) to evaluate the treatment (approach), the answer to $Q_\textsc {nde}$ is known as average nde.

**Definition 3**

**(****nde****)** The nde of a binary treatment $T$ on an outcome $Y$ mediated by $X$ is given by

\begin{equation*}
\textsc {nde}_{0\rightarrow 1} = \mathbb {E}[Y_{X(0)}|do(T=1)] - \mathbb {E}[Y|do(T=0)] \tag{3}
\end{equation*}

where $X(0)$ refers to the counterfactual of $X$ had $T$ been 0.

### E. Natural Indirect Effect (nie)

For isolating the indirect effect that a country exhibits on case fatality only via age, $C\rightarrow A\rightarrow F$, we run into the additional complication that it is not possible to keep the influence along $C\rightarrow F$ constant under a change in country. To overcome this problem, one can consider a hypothetical change in the distribution of the mediator (age) as if the treatment (country) were changed, but without actually changing it. E.g., we may ask

$Q_\textsc {nie}$: *“How would the overall cfr in China change if the case demographic had instead been that from Italy while keeping all else (i.e.,* cfr*'s of each age group) the same?”*

Since this considers a change of the mediator (age) to the natural distribution it would follow under a change treatment (case demographic from Italy) while keeping the treatment the same (Chinese cfr's), the answer to this question is referred to as the average nie.

**Definition 4**

**(****nie****)** The nie of a binary treatment $T$ on an outcome $Y$ with mediator $X$ is given by

\begin{equation*}
\textsc {nie}_{0\rightarrow 1} = \mathbb {E}[Y_{X(1)}|do(T=0)] - \mathbb {E}[Y|do(T=0)]. \tag{4}
\end{equation*}

### F. Mediation Formulas

For causally sufficient systems, the interventional distributions of each variable given its causal parents equal the corresponding observational distributions, reflecting the intuition that they represent *mechanisms* rather than mere mathematical constructs [19]. tce (1) and cde (2) then reduce to

\begin{align*}
\textsc {tce}^{\text{obs}}_{0\rightarrow 1} =& \mathbb {E}[Y|T=1] - \mathbb {E}[Y|T=0]\tag{5} \\
\textsc {cde}^{\text{obs}}_{0\rightarrow 1}(x) =& \mathbb {E}[Y|T=1, X=x] - \mathbb {E}[Y|T=0, X=x]. \tag{6}
\end{align*}

Moreover, in this case, nde (3) and nie (4) are given by the following *mediation formulas* [18]:

\begin{align*}
\textsc {nde}^{\text{obs}}_{0\rightarrow 1} =& \textstyle \sum \limits_{x}P\left(X=x|T=0\right)\left(\mathbb {E}[Y|T=1, X=x] \right. \\
& \left. - \mathbb {E}[Y|T=0, X=x]\right)\tag{7} \\
\textsc {nie}^{\text{obs}}_{0\rightarrow 1} =& \textstyle \sum \limits_{x} \left(P(X=x|T=1) \right. \\
&\left. -P(X=x|T=0)\right)\mathbb {E}[Y|T=0, X=x]. \tag{8}
\end{align*}

When comparing cfrs across countries, we only have observational data and, thus, rely on causal sufficiency (see III) to compute total, direct, and indirect effects via (5)–(8).

### G. Relation Between tce, nde, and nie

*Can the TCE be decomposed into a sum of direct and indirect contributions?* While such an additive decomposition indeed exists for linear models, it does not hold in general due to possible interactions between treatment and mediator, referred to as *moderation*.^4^^4^Pearl and Mackenzie[20] give the illustrative example of a drug (treatment) that works by activating some proteins (mediator) inside the body before jointly attacking the disease: the drug is useless without the activated proteins (so the direct effect is zero) and the activated protein is useless without the chemical compound of the drug (so the indirect effect is also zero), but the total effect is nonzero because of the interaction between the two. Direct and indirect effects are not uniquely defined in general, but depend on the value of the mediator. Counterfactual quantities, such as nde and nie, are thus useful tools to measure some average form of direct and indirect effect with a meaningful interpretation.

### H. Mediation Analysis in AI: Algorithmic Fairness

While the present work is focused on the study of COVID-19 cfrs, we remark that the ideas and tools of causal mediation analysis presented in this section also feature prominently in other areas of AI, e.g., in the field of algorithmic fairness, which aims to uncover and correct for discriminatory biases of models. In this context, discrimination is often interpreted as a causal influence of a protected attribute (such as age, sex, ethnicity, etc.) on an outcome of interest along paths that are considered unfair for a setting at hand [21]–[25].

A historic example and a famous instance of Simpson's paradox is the case of UC Berkeley graduate admissions [26]: in 1973, pooled data across all departments showed that a substantially larger proportion of all male applicants were admitted (44%) when compared to females (35%), suggesting gender bias. However, careful mediation analysis subsequently revealed that this difference was entirely explained by the choice of department—females generally applied to departments with lower admission rates—and that when controlling for the mediating variable “department choice,” i.e., considering the *direct effect* of sex on admission, there was actually a small bias *in favor* of women [26]. Since the *indirect path* mediated by department choice was not considered unfair for the admission process, no wrongdoing on behalf of the school was concluded.

## V. Case Study: Mediation Analysis of Age-Related Effects on COVID-19 cfrs

### A. Dataset

To employ the tools from mediation analysis outlined in IV to better understand the influence of age on COVID-19 cfrs, we curated a dataset of confirmed cases and fatalities by age group (0–9, 10–19, etc.) from 11 countries (Argentina, China, Colombia, Italy, the Netherlands, Portugal, South Africa, Spain, Sweden, Switzerland, and South Korea) and the *Diamond Princess* cruise ship, on which the disease spread among passengers forced to quarantine on board [27]. The dataset includes 756 004 cases and 68 508 fatalities (total cumulative cfr of 9.06%), reported either by the different countries’ national health institutes or in scientific publications. The selection of countries is based on availability of suitable data at the time of writing.^5^^5^Unfortunately, conventions on how to group patients by age vary across countries: e.g., Belgium, Canada, France, and Germany do not consistently use ten-year intervals; others, such as the US, use different groupings (0–4, 5–14, etc.). For some countries (e.g., Brazil, Russia, Turkey, and U.K.), we did not find demographic data. Where available, we included several reports from the same country, e.g., for Italy and Spain in weekly intervals. The data and our analysis (in form of an interactive notebook) are provided in the supplement and will be made publicly available. The exact sources and several additional figures and tables can be found in Appendices E and F.

### B. Tracing Causal Effects Over Time

**Fig. 3.**
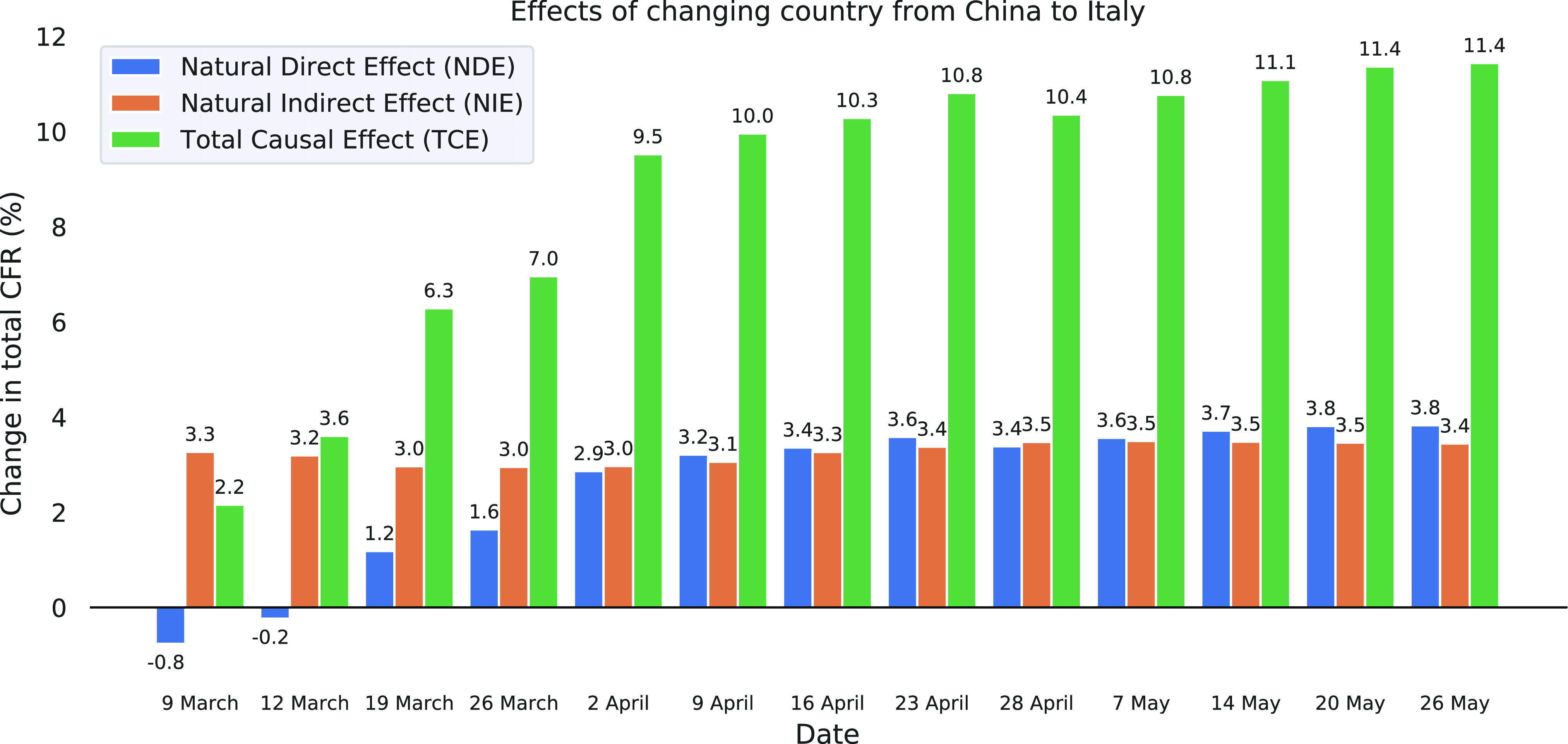
Evolution of tce, nde, and nie of changing country from China to Italy on total cfr over time. We compare static data from China [4] with different snapshots from Italy reported in [5]. The direct effect initially was negative, meaning that age-specific fatality in Italy was lower; however, it changes sign around mid-March when an overloaded health system in northern Italy was reported [3].

First, we investigate the temporal evolution of direct and indirect (age-mediated) causal effects on fatality by expanding on the comparison from II. The result of tracing tce, nde, and nie of changing from China to Italy over a period of 11 weeks using (approximately) weekly reports from the work in [5] is shown in Fig. 3. Note that case and fatality numbers for China remain constant in the figure, so any changes over time can be attributed to Italy.^6^^6^At the time of writing, not many new cases have been reported from China since the study of Wu and McGoogan [4].

We find that the tce—which measures what would happen to the total cfr if *both* cfrs by age group *and* case demographic were changed to those from Italy—is positive throughout, reflecting a higher total cfr in Italy. It increases rapidly from an initial 2.2% to 9.5% over the first three weeks considered, and then continues to rise more slowly to 11.4%. This indicates that the difference between the two countries’ total cfr becomes more pronounced over the time. In order to understand what drives this difference, we next consider the direct and indirect effects separately.

The nde—which captures what would happen to the total cfr if the case demographic was kept the same, whereas only the approach (cfrs per age group) was changed— is negative at first, meaning that the considered change in approach would initially be beneficial, consistent with the lower cfrs in each age group shown in Fig. 1. However, at a turning point around mid-March, the nde changes sign: beyond this point, switching to the Italian approach would lead to an increase in total cfr. While we can only speculate about the precise factors that came together in producing this reversal in nde, it seems worth pointing out that an overwhelmed healthcare system “close to collapse” in (northern) Italy was reported during that very period of early to mid-March [3]. The nde then keeps rising steeply until April before gradually flattening off, similar to the tce.

The nie—which measures what would happen to total cfr if the approach was kept the same, whereas the case demographic was changed to that in Italy—on the other hand remains largely constant over time, fluctuating between 3% and 3.5%, indicating that the case demographic in Italy does not change much over time. Its large value of over 3% means that simply changing the case demographic from China to that in Italy would already lead to a substantial increase in total cfr, consistent with the larger share of confirmed cases amongst the elderly in Italy shown in Fig. 1.

In summary, *while indirect age-related effects considerably contribute to differences in total CFR*—especially initially, when the instance of Simpson's paradox from II is reflected in the opposite signs of nde and nie—*it is mainly the direct effect that drives the observed changes over time.*

### C. Comparison Between Several Different Countries

**Fig. 4.**
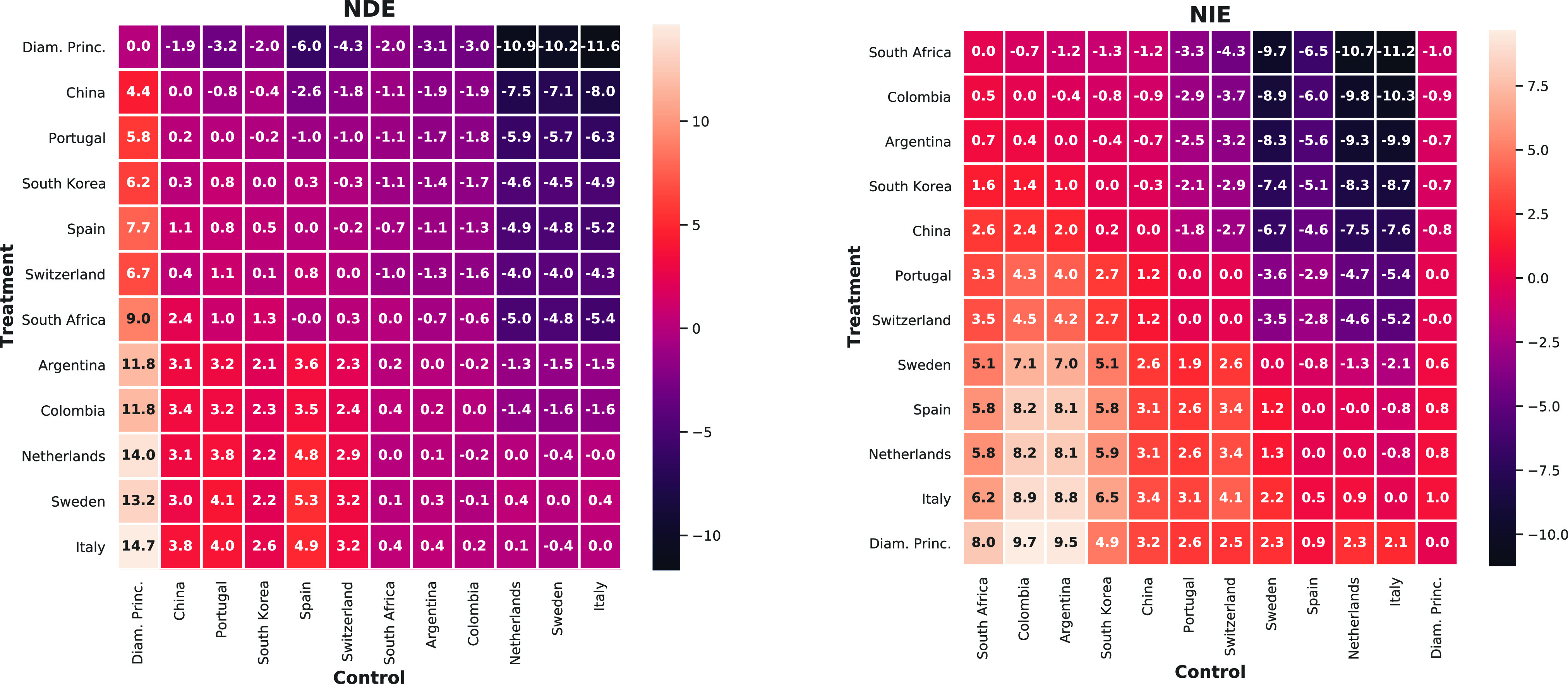
ndes (left) and nies (right) for switching from the control country (columns) to the treatment country (rows). Numbers show the change in total cfr in percentage, i.e., negative numbers indicate that switching to the treatment country's approach, i.e., its cfrs by age group, (nde) or case demographic (nie) would lead to a decrease in total cfr. Countries are ordered by their average effect as a treatment country (nde or nie) over the remaining 11 data points as a control.

We now leave the specific example of China versus Italy aside and turn to a comparison of causal effects between the 12 countries (including the Diamond Princess) contained in our dataset. All pairwise effects on total cfr (in %) of changing only *“approach,”* i.e., the cfrs by age group, (nde; left) or case demographic (nie; right) from a control country (columns) to a treatment country (rows), are shown in Fig. 4.

For ease of visualization, the order in which countries are presented in Fig. 4 was chosen according to their average effect as a treatment over the remaining countries as control (i.e., by the mean of rows) for nde and nie separately. This allows to read off trends about the effectiveness of different approaches and the influence of the case demographic (subject to limitations, such as, e.g., differences in testing, which we discuss further in VI). In the case of nde, for example, the Diamond Princess, China, Portugal, and South Korea compare favorably to most others in terms of their approaches, whereas the Netherlands, Sweden, and Italy occupy the bottom end of the range. In the case of nie, South Africa, Colombia, and Argentina benefit most from their case demographic, whereas Spain, the Netherlands, Italy, and the Diamond Princess are particularly adversely affected by it.

Notably, there is no significant correlation between countries’ ranking by nde and nie (Spearman's $\rho =0.04$ and $p=0.9$), suggesting that a country's approach and case demographic may be largely unrelated. While some countries, such as South Korea, Switzerland, the Netherlands, and Italy, take almost the same place according to both rankings of particular interest are those countries for which rankings by nde and nie differ most. Other than for the Diamond Princess—which due to small sample size and high testing rates constitutes an illustrative special case that we discuss further in VI—the case of high ranking (rk) in terms of nde and low ranking in terms of nie is most pronounced for Spain ($\text{rk}_\textsc {nde}-\text{rk}_\textsc {nie}=-4$), Portugal ($-3$), and China ($-3$). This suggests that for the case of Spain, the high total cfr may, at least in parts, be attributed to an unfavorable case demographic, whereas the approaches (age-specific fatality) of China and Portugal may be even better than suggested by their (already comparatively low) total cfrs. Conversely, countries that rank considerably higher in terms of nie than nde include Colombia ($+7$), South Africa ($+6$), and Argentina ($+5$). These countries’ low total cfrs may thus wrongly suggest a very successful approach while the low total cfr may actually, at least in parts, be due to an advantageous case demographic—again, subject to caveats, such as differences in testing, see VI for more details.

Noting that South Africa, Colombia, and Argentina are also the three youngest amongst the considered countries in terms of median age, we computed the Spearman correlation between the ranking of countries by nie and by their median age and found a strong correlation between the two ($\rho =0.94, p=7\times 10^{-6}$). This indicates that for the countries considered, the case demographic is predominantly determined by the age distribution of the population, and suggests that countries seem not to make (effective) use of strategies, such as, e.g., age-specific quarantines.

**Fig. 5.**
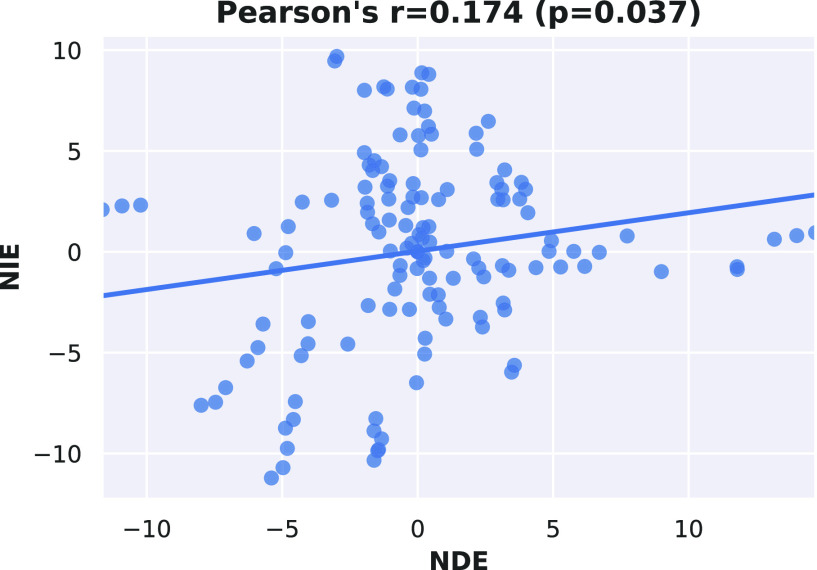
Scatter plot of nie versus nde between all 132 pairs of distinct countries: we find a weak but statistically significant positive correlation (see plot title).

As a further investigation into the relation between direct and indirect effects on COVID-19 fatality, we find that, of the 132 ordered pairs of distinct countries, 64 exhibit opposite signs of nde and nie (as for the example of Simpson's paradox in II, see also dates from early March in Fig. 3), meaning that *comparing countries in terms of total* cfr* may not give an accurate picture of the relative effectiveness of two countries’ approaches* in those cases. Overall, pairwise ndes and nies are only weakly but significantly correlated (Pearson's $r=0.17, p=0.04$), as shown in Fig. 5.

## VI. Limitations and Future Work

In this article, we have taken a coarse-grained causal modeling perspective considering the variables country $C$, age group $A$, and case fatality $F$, which are reported in the context of COVID-19 cfr data. This view abstracts away many potentially important factors (some of which we named in III) along the paths of the assumed causal graph. A strength of this approach is that it allows for consistent reasoning about age-mediated and nonage-related effects within the assumed model in situations where the data does not support a more fine-grained analysis. On the other hand, any conclusions must be interpreted within this coarse-grained view: we have thus collectively referred to various country-specific influences on fatality as “approach.”

### A. Considering Additional Mediators

**Fig. 6.**
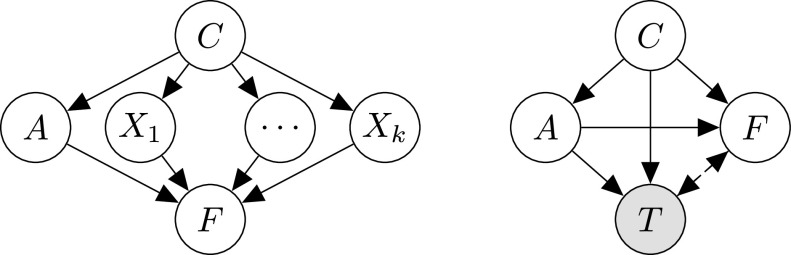
(Left) Direct effect $C\rightarrow F$ is likely mediated by additional variables $X_i$. (Right) Testing strategy may introduce selection bias, since cfr data implicitly conditions on having tested positive, represented by the shaded $T$.

It is safe to assume that the virus is ultimately agnostic to the notion of different “*countries*” and that the influence of country on fatality $C\rightarrow F$ is not actually a direct one, but instead mediated by additional variables $X_i$, as illustrated in Fig. 6 (left). Candidates for such additional mediators $X_i$ include, e.g., nonpharmaceutical interventions and critical healthcare infrastructure. We believe that many questions of interest regarding the COVID-19 pandemic can be phrased as path-specific causal effects involving such mediators, e.g., *“What would be the effect on total* cfr* if country $C_1$ bought as many ventilators as country $C_2$?”* Assuming more fine-grained data will become available as the pandemic progresses, extending our model with additional mediators and investigating their effects by building on the tools described in IV is a promising future direction to deepen our understanding about which factors most drive COVID-19 fatality.

### B. Testing Strategy and Selection Bias

An important potential limitation of our approach (or, more fundamentally, of cfr data) is that we only consider confirmed cases, i.e., patients who tested positively for COVID-19. We can make this explicit in our model by including test status $T$ as additional variable. Our data are then always conditioned on $T=1$, as illustrated in Fig. 6 (right). Since who is tested is not random, but generally depends both on a country's testing strategy and a patient's age (e.g., via severity of symptoms), reflected by the arrows $\lbrace C,A\rbrace \rightarrow T$, this results in a problem of *selection bias* [28]. This issue is particularly clear for the Diamond Princess on which *“3063 PCR tests were performed among the* [3711] *passengers and crew members. Testing started among the elderly passengers, descending by age”* [27]. As a result of such extensive testing, the proportion of asymptomatic cases on board was very high (318 out of 619 detected cases), leading to low cfrs as manifested in the negative ndes for the Diamond Princess as treatment in Fig. 4. This rate of testing is presently not feasible for countries with millions of inhabitants. Since testing capacities differ across countries, the reported cfrs may thus often not be comparable. Building on recent (causal) work on recoverability from selection bias may help address this aspect of the problem [29], [30].

A second source of bias may stem from the choice of countries included in our dataset: we only considered countries that report age-stratified cfrs—those might be particularly affected by the pandemic. The cumulative cfr of 9% is thus likely inflated by such selection processes. An additional problem is the delay between time of infection and death: to correct for this, fatalities should be divided by the number of patients infected at the same time as those who died, i.e., excluding the most recent cases  [31], which requires estimating the incubation period.

### C. cfr Versus *Infection* Fatality Rate (ifr)

To overcome such testing and delay issues, one should ideally instead use the (delay-corrected) ifr, defined as the ratio of fatalities over *all infected patients, including asymptomatic ones*. However, this requires estimating the number of undetected cases based on specific modeling assumptions (which may not hold in practice, thus potentially introducing additional biases) for each country or region separately, and consequently, we are only aware of very few estimates of age-stratified ifrs (e.g., [27], [32], [33]). Our analysis may be adapted for ifr data as well though, see Appendix VIII-C for more details.

## VII. Discussion

The problem of cfrs is a compelling example of Simpson's paradox, which brings to bear a core method of AI (causal reasoning) on a COVID-19 problem. We would like to place this in a broader context by discussing additional links between Simpson's paradox and AI, and contributions of AI in the ongoing pandemic.

### A. Simpson's Paradox in the Context of AI

We have aforementioned examples of Simpson's paradox in college admission policies [26] and epidemiology [10]. In addition, it has been observed that the paradox may occur in many other real-life contexts [34], [35], thus making its understanding relevant to the field of artificial intelligence, commonsense reasoning, and in the study of uncertain reasoning systems in general. Furthermore, the reversal in Simpson's paradox becomes critical in decision making situations [12], [36], where an agent needs to move beyond a merely predictive setting and reason about the effect of actions or interventions. As already discussed, the paradox can be “resolved” in different ways depending on the causal model (e.g., whether covariates take the role of confounders or mediators) and the causal query of interest to the agent (e.g., whether a direct, indirect, or TCE is to be estimated). If variables that are relevant for a correct resolution of the “paradox” are not directly observed, this can be particularly problematic, and causal reasoning therefore bears nontrivial conceptual and algorithmic implications, e.g., in sequential decision making contexts, such as the multiarmed bandit problem (see [37], [38]).

Since Simpson's paradox demonstrates that opposite conclusions can be reached depending on how the data are aggregated or stratified, it also has close connections to clustering [39], [40], another core AI technique, which is especially challenging for high-dimensional data as is commonplace in the age of big data. Other seemingly paradoxical reversals, related to Simpson's paradox, can also occur in the context of games; for example, in Parrondo's paradox, a coin flip game with a positively biased outcome can be generated from the combination of two negatively biased processes [41], [42].

### B. AI Against COVID-19: A Causal View

Given the global disruption caused by COVID-19, there is a growing body of work trying to leverage AI and data science to help curtail and combat the ongoing pandemic, e.g., in contact tracing [43], [44], symptom screening [45], risk scoring [46], vaccine development [47], or diagnosis from CT [48] or X-ray [49] imaging—see, e.g., [50]–[52] for reviews. Due to typically small sample sizes and population differences, however, such applications of AI need to be critically assessed with respect to transparency and generalizability to different cohorts of individuals [53]. Indeed, a recent meta-analysis of 232 models for diagnosis, prognosis, and detection of COVID-19 concluded that “almost all published prediction models are poorly reported, and at high risk of bias such that their reported predictive performance is probably optimistic” [54].

The question whether a machine learning model will generalize outside its training distribution is closely linked to some of the concepts from causality discussed in this article and has been studied in the causal inference literature under the term “transportability” [55], [56]. If, as is common practice, the aim is to maximize predictive performance on the available data, then any trained model is encouraged to rely on “spurious” correlations (e.g., due to unobserved confounding), which may not generalize to different populations (e.g., different countries) or modes of reasoning, such as reasoning about the outcome of treatment interventions based on observational data. Causal mechanisms, on the other hand, constitute stable (or invariant) units, which are often largely independent of other components of a system and should thus be transferable even if the distribution of some features changes [19], [57]. The aforementioned reasoning cautions against blind use of supervised learning techniques without regard to the underlying causal structure. Indeed, we would argue that applications of AI techniques on COVID-19 may often benefit from formulating a causal model underlying the observed data (including potential population differences), as done in some studies [58]–[60].

## VIII. Conclusion

We have shown how causal reasoning can guide the interpretation of COVID-19 case fatality data. In particular, mediation analysis provides tools for separating effects due to different factors, which, if not properly identified, can lead to misleading conclusions. We exploited these tools to uncover patterns in the time evolution of cfrs in Italy, and in the comparison of multiple countries. To study age-mediated and age-unrelated effects on cfr across different countries, we curated a large-scale dataset from a multitude of sources.

## Supplementary Materials

Supplementary Materials.

AGE-STRATIFIED COVID-19 CASE FATALITY RATES (CFRS): DIFFERENT COUNTRIES AND LONGITUDINAL
