# Supplementary material for: Simpson's Paradox in COVID-19 Case Fatality Rates: A Mediation Analysis of Age-Related Causal Effects
Source: IEEE Trans Artif Intell. 2021 Apr 14;2(1):18–27. doi: 10.1109/TAI.2021.3073088 (PMC8791436; doi:10.1109/TAI.2021.3073088)
Supplement: AGE-STRATIFIED COVID-19 CASE FATALITY RATES (CFRS): DIFFERENT COUNTRIES AND LONGITUDINAL [file tai-3073088-mm.zip › tai-3073088-mm/appendix.pdf]

APPENDIX

A. Additional concepts from mediation analysis

1) Experimental (non-)identifiability of direct and indirect effects: Since the CDE in (2) only involves interventional quantities it is in principle *experimentally identifiable*, meaning that it can be determined through an experimental study in which both the treatment and the mediator are randomised, thus providing valid estimates of $P(Y|do(T=t, X=x))$.

In contrast, NDE and NIE are, *in general* (i.e., without further assumptions), *not experimentally identifiable* owing to their counterfactual nature. However, under certain conditions such non-confoundedness of mediator and outcome experimental identifiability is obtained.[7] In this case:

$$\text{NDE}^{\text{exp}}_{0\to1} = \textstyle\sum_x P\big(X=x|do(T=0)\big)\left(\mathbb{E}[Y|do(T=1, X=x)] - \mathbb{E}[Y|do(T=0, X=x)]\right),$$
$$\text{NIE}^{\text{exp}}_{0\to1} = \textstyle\sum_x \big(P\big(X=x|do(T=1)\big) - P\big(X=x|do(T=0)\big)\big)\,\mathbb{E}[Y|do(T=0, X=x)]\,.$$

Note that even then, identifying natural effects requires combining results from two different experimental settings: one where both mediator and treatment are randomised, and a second in which treatment is randomised and the mediator observed. This again highlights the hypothetical nature of NDE and NIE and explains why they—unlike TCE and CDE—cannot simply be read off from a table like Table III, even when causal sufficiency is assumed.

2) Subtractivity principle: There exists a general formula relating TCE, NDE, and NIE known as the *subtractivity principle* that follows from their definitions and holds without restrictions on the type of model [18]:

$$\text{TCE}_{0\to1} = \text{NDE}_{0\to1} - \text{NIE}_{1\to0} = \text{NIE}_{0\to1} - \text{NDE}_{1\to0}.$$

B. Example calculations for TCE, CDE, NDE and NIE

1) TCE: To address Q_{TCE} in our example we need to compute

$$\text{TCE}_{\text{China}\to\text{Italy}} = \mathbb{E}[M|do(C=\text{Italy})] - \mathbb{E}[M|do(C=\text{China})]. \tag{9}$$

From the assumed causal graph and causal sufficiency, it follows that for our setting $P(A|do(C)) = P(A|C)$ and $P(M|do(A,C)) = P(M|A,C)$. We can thus compute (9) as

$$\begin{aligned}\text{TCE}_{\text{China}\to\text{Italy}} &= \sum_a \left[P_{M|A,C}(1|a,\text{Italy})P_{A|C}(a|\text{Italy}) - P_{M|A,C}(1|a,\text{China})P_{A|C}(a|\text{China})\right]\\ &\approx 2.2\%.\end{aligned}$$

Note that this corresponds to the difference of total CFRs reported in the last column of Table III. This means that the difference of total CFRs indeed constitutes a causal effect, and changing country from China to Italy would lead to an overall increase in CFR of $\approx 2.2\%$ (given the data in Table III and subject to our modelling assumptions).

2) CDE: To address $Q_{\text{CDE}(a)}$ in our example, we need to compute

$$\begin{aligned}\text{CDE}_{\text{China}\to\text{Italy}}(a) &= \mathbb{E}[M|do(C=\text{Italy}, A=a)] - \mathbb{E}[M|do(C=\text{China}, A=a)]\\ &= P(M=1|do(C=\text{Italy}, A=a)) - P(M=1|do(C=\text{China}, A=a))\\ &= P(M=1|C=\text{Italy}, A=a) - P(M=1|C=\text{China}, A=a).\end{aligned}$$

This corresponds to the difference between CFRs across the two countries within a particular age group, i.e., the difference of two CFRs within a particular column of Table III. Hence, the answer to $Q_{\text{CDE(50–59)}}$ is that for this age group it is safer to switch country to Italy with a resulting change in CFR of $\approx 0.2\% - 1.3\% = -1.1\%$. (Bear in mind that this calculation is based on Italian data from beginning of March.)

3) NDE: Applying our assumptions, in particular causal sufficiency, we can calculate the NDE to answer Q_{NDE} for our running example as follows,

$$\begin{aligned}\text{NDE}_{\text{China}\to\text{Italy}} &= \mathbb{E}[M_{A(\text{China})}|do(C=\text{Italy})] - \mathbb{E}[M_{A(\text{China})}|do(C=\text{China})]\\ &= \sum_a P_{A|do(C)}(a|do(\text{China}))\left[P_{M|do(A,C)}(1|do(a,\text{Italy})) - P_{M|do(A,C)}(1|do(a,\text{China}))\right]\\ &= \sum_a P_{A|C}(a|\text{China})\left[P_{M|A,C}(1|a,\text{Italy}) - P_{M|A,C}(1|a,\text{China})\right]\\ &= \mathbb{E}_{A|C=\text{China}}\left[\text{CDE}_{\text{China}\to\text{Italy}}(A)\right] \approx -0.8\%.\end{aligned}$$

[7] A more general criterion is the existence of a set of covariates W, non-descendants of T and X, which satisfy the graphical d-separation criterion $(Y \perp\!\!\!\perp X|W)_{G_{\underline{\text{TX}}}}$, see [18, Thms. 1&4] for details.

We thus find that when we only consider the Chinese case demographic, using the Italian approach (i.e., the CFRs for Italy from Table III) would lead to a reduction in total CFR of $\approx 0.8\%$, consistent with our observation from §II that CFRs were lower in Italy for each age group.

Remark 1. *As is apparent from the last line of the above calculation, the* NDE *can be interpreted as an expected* CDE *w.r.t. a particular (counterfactual) distribution of the mediator. Here, due to our assumption of causal sufficiency the expectation is taken w.r.t. the conditional distribution of A in the control group (China).*

Remark 2. *Taking the previous remark about* NDE *as the expected* CDE *within the control group one step further, we can, of course, also consider expected* CDEs *w.r.t. other distributions describing a target-population we want to reason about. For example, a third country, say Spain, may be considering whether to adopt the Chinese or Italian approach given its own case demographic. In this case, we would be interested in the following quantity.*

$$\mathbb{E}_{A|C=Spain}[\text{CDE}_{China\to Italy}(A)] = \sum_a P_{A|C}(a|Spain)\text{CDE}_{China\to Italy}(a)$$

4) NIE*:* Again, using causal sufficiency, we can calculate the NIE to answer Q_{NIE} for our example as follows,

$$\begin{aligned}\text{NIE}_{\text{China}\to\text{Italy}} &= \mathbb{E}[M_{A=A_{\text{Italy}}}|do(C=\text{China})] - \mathbb{E}[M_{A=A_{\text{China}}}|do(C=\text{China})] \\ &= \sum_a \left[P_{A|do(C)}(a|do(\text{Italy})) - P_{A|do(C)}(a|do(\text{China}))\right] P_{M|do(A,C)}(1|do(a,\text{China})) \\ &= \sum_a \left[P_{A|C}(a|\text{Italy}) - P_{A|C}(a|\text{China})\right] P_{M|A,C}(1|a,\text{China}) \\ &\approx 3.3\%\end{aligned}$$

We thus find that changing only the case demographic to that from Italy would lead to a substantial increase in total CFR in China of about 3.3%. Notably, the NIE is of the opposite sign of the NDE suggesting that indirect and direct effects are counteracting in our example as the reader may have expected from §II: despite the lower CFRs in each age group (leading to a negative NDE) the total CFR is larger in Italy due to the higher age of positively-tested patients (leading to a positive NIE).

5) Substractivity-principle: In our running example we find that

$$\text{TCE}_{\text{China}\to\text{Italy}} = 2.2\% \neq -0.8\% + 3.3\% = \text{NDE}_{\text{China}\to\text{Italy}} + \text{NIE}_{\text{China}\to\text{Italy}}$$

indicating that some level of moderation or interaction is present.

*C. Case Fatality Rate (*CFR*) vs Infection Fatality Rate (*IFR*)*

As discussed in §VI, the number of confirmed cases (i.e., the denominator in the CFR) in any given country strongly depends on the testing strategy the country implements, and could be affected by multiple sources of selection bias. This can potentially limit the scope of conclusions drawn based on the reported CFRs.

An alternative measure is the *Infection Fatality Rate* (IFR), which represents the proportion of fatalities among all infected individuals—including all asymptomatic and undiagnosed subjects. Due to limited testing capacity, testing randomly selected subpopulations (irrespective of symptoms) to get an accurate picture of the number of true infections is usually infeasible—at least during early stages of a pandemic. Consequently, the IFRneeds to be estimated, which can be difficult as it relies on elusive and often unobserved quantities. Under suitable assumptions and with additional data and epidimiological background knowledge, however, it may be inferred using a model-based approach [32, 33]. Additionally, in some cases it can be estimated from large scale serological surveys. We will briefly describe these two approaches in C1 and C2

As a first remark, we note that IFR data suitable for a large scale study involving a comparison between multiple different countries and at different points in time as presented in this paper is difficult to find; model-based estimation could on the other hand be incorporated in our framework, as we detail below, but it is subject to assumptions which might in some cases be questionable.

We additionally want to stress that the causal model we propose in this work, and specifically in §III, could be applied to CFR and IFR data alike: crucially, even if it were possible to perfectly estimate IFRs, causal modeling would still be required for its interpretation [61], and mediation analysis would still provide a useful tool for comparing different countries based on that measure and separating and quantifying age-mediated and non-age-related contributions, similarly to what we have shown in this work for CFRs.

1) Model-based estimation of the Infection Fatality Rate: [32] proposed a model-based approach to correct the reported CFRs by combining different adjustments to obtain an estimate of the IFR. They performed such estimation for the case of Wuhan, China, which we summarise below.

The first step is an estimation of the interval between the onset of symptoms and death (or discharge from hospital) for infected patients. This is obtained with a combination of observational data (where available) and model-based imputation of the onset of symptoms of hospitalized patients. Note that in estimating time intervals between symptom onset and outcome, it

was necessary to account for the fact that, during a growing epidemic, a higher proportion of the cases will have been infected recently, thereby requiring an adjustment for the epidemic growth.

The reported CFRs are then corrected based on the population demographic, by assuming that the *attack rate* — the proportion of people who become ill with a disease in a population initially free of the disease — is homogeneous across the different age groups. Under this assumption, the demographic distribution of cases by age across each location should broadly match the demography of the populations in Wuhan and across the rest of China—note that this assumption might become problematic when inter-generational mixing patterns are not homogeneous across age ranges, thus favoring the spreading of the disease within specific age ranges [62]. By further assuming complete ascertainment in the age-group where the attack rate is highest—-that of the 50–59 year olds in this example—the authors can then adjust cases in the other age groups to produce identical attack rates. Further underreporting of positive cases is estimated based on data of international residents who were repatriated from Wuhan.

A statistical model is proposed to jointly fit the age-stratified adjusted case-fatality ratio, the onset-to-death distribution and the true underlying number of cases within Wuhan and other areas of mainland China. The IFR is then estimated based on these quantities.

Beyond the details and specific modeling choices operated by [32], we remark that this model-based estimation can be simply integrated within our causal investigation, by substituting the the reported number of cases used for CFRs by the estimated number of infections under the specified model to obtain IFRs.

To additionally address the concern that official death counts could also be underestimated in some cases, [33] collected demographic and death records data from the Italian Institute of Statistics; focusing on the area in Italy that experienced the initial outbreak of COVID-19, they estimated a Bayesian model fitting age-stratified mortality data from 2020 and previous years. This allowed them to build more reliable estimates of the total death count.

2) Estimation based on seroprevalence surveys: In some cases, testing and seroprevalence surveys involving the vast majority of the population of a given region can also provide reliable estimates of the IFR. One such case which we already reported and extensively discussed in §VI is that of the cruising ship *Diamond Princess* [27], where almost all passengers where tested, and, as a consequence, there is essentially no difference between the reported CFR and IFR.

[63] present an estimation of IFRin Lombardy, Italy, based on cases identified via contact tracing between February and April 2020, additionally complementing these data with the results of a serological survey started on the 16th of April 2020. A similar serology-informed study was conducted in Geneva, Switzerland [64]. Note that different age stratification in [64] and [63] makes a direct comparison tricky, a problem we already encountered for CFR data, see footnote 4 in §V.

3) IFR *in Lombardy before and after the 16th of March, 2020:* For completeness, we report an example computation of the different causal effects discussed in the paper with IFR data from Lombardy, Italy, based on [63], as discussed above. This data is shown in Table II. In Table I we report the TCE, NDE, and NIE computed assuming data from pre-16th of March Lombardy as a baseline and post-16th of March as a treatment. Note that by the nature of the IFR, the number of fatalities and infections included in these two periods are mutually exclusive, so that double counting is not an issue.

We find that the TCE is negative, reflecting a lower IFR after the 16th of March. Moreover, the NDE is also negative, which seems to suggest that the change in *approach* would be beneficial, while the NIE is positive, reflecting a slight shift towards a less favourable infection demographic after the 16th of March.

Overall, these results might reflect an improvement of the doctors' ability to treat the disease over time as more and more experience regarding the virus was being gathered. The observed trend might also be related to the already mentioned overload of the Italian healthcare system [3] in the early phase of the epidemic. However, due to different time-delay effects in the IFR and CFR, it is possible that the infection peak differs among these two measures. Furthermore, this regional data is not directly comparable to the nation-wide data presented in §V: while Lombardy has probably been one of the main drivers of the evolution of the epidemic in Italy, since it was one of the hardest hit regions [65], the aggregated national data also reflects the dynamics of different regions where infection peaks might have been attained at a later time. In general, we stress that the comparison of these two metrics is thus highly nontrivial and requires incorporating additional knowledge on the dynamics of the epidemic.

TABLE I: Data from Lombardy [63]; causal effects when switching from before to after the 16th of March. The baseline is based data from before 16th of March, while the treatment consists of data from after 16th of March.

TCE	NDE	NIE
-1.63%	-1.57%	0.18%

TABLE II: Infection fatality rates in Lombardy.

Age	0–19	20–49	50–59	60–69	70–79	$\geq$ 80
Overall (n=5,484)	0.0% (0/304)	0.0% (0/885)	0.46% (3/648)	1.42% (7/494)	6.87% (23/335)	18.35% (29/158)
Before the 16 March 2020 (n=2,696)	0.0% (0/114)	0.0% (0/438)	0.56% (2/354)	1.54% (4/259)	7.94% (15/189)	30.43% (21/69)
After the 16 March 2020 (n=2,721)	0.0% (0/188)	0.0% (0/431)	0.35% (1/283)	0.88% (2/227)	5.59% (8/143)	8.14% (7/86)

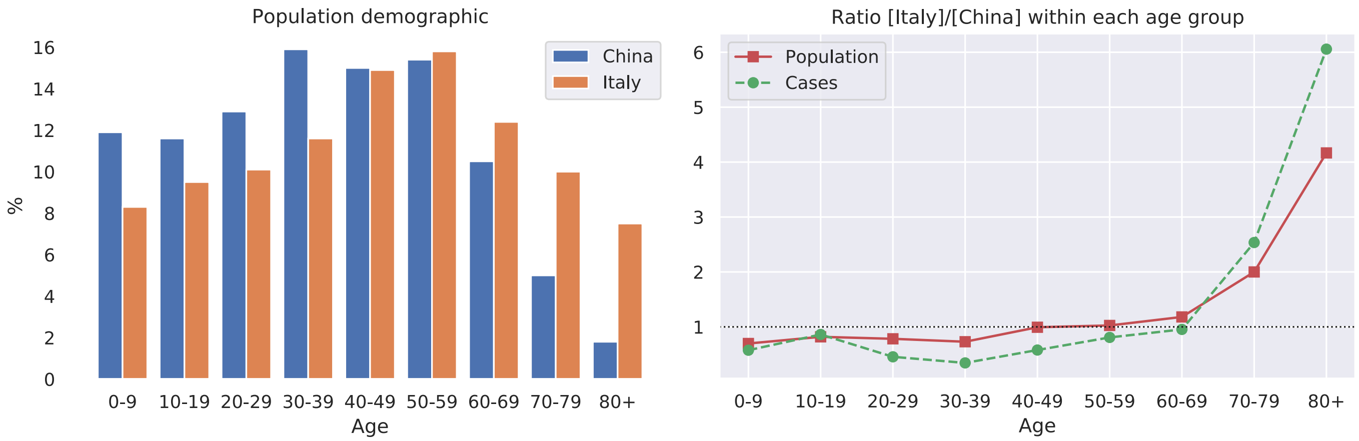

(a) Population demographic

(b) Ratios

Fig. 7: Visualisation of the data from Tables IV and V for the demographic comparison of China and Italy. (a) Demographic of the general population in the two countries (c.f. Fig. 1). (b) Ratios (Italy / China) of the proportion of confirmed cases by age group (shown in dashed green) and the proportion of the general population within each age group from Table V (shown in solid red).

D. *Further material on the comparison China vs. Italy*

In this Appendix, we provide additional details on the comparison of Italy and China that gives rise to the instance of Simpson's paradox in §II and that was further investigated with a longitudinal approach in Fig. 3. Tables III, IV, and V show the CFRs, case demographic, and demographic of the general population for the two countries, respectively. The relationship between case demographic and demographic of the general population is further investigated and visualised in Fig. 7. Fig. 8 shows the temporal evolution of age-specific CFRs and case demographic for the longitudinal data from Italy used in Fig. 3.

TABLE III: Exact numbers for the comparison of case fatality rates (CFRs) by age group for Italy and China shown in Fig. 1. Absolute numbers of fatalities/confirmed cases are shown in brackets below. Lower CFRs are highlighted in bold face. Sources: [4] and [5].

Age	0–9	10–19	20–29	30–39	40–49	50–59	60–69	70–79	$\geq$ 80	Total
Italy	**0%** (0/43)	**0%** (0/85)	**0%** (0/296)	**0%** (0/470)	**0.1%** (1/891)	**0.2%** (3/1,453)	**2.5%** (37/1,471)	**6.4%** (114/1,785)	**13.2%** (202/1,532)	4.4% (357/8,026)
China	**0%** (0/0)	0.2% (1/549)	0.2% (7/3,619)	0.2% (18/7,600)	0.4% (38/8,571)	1.3% (130/10,008)	3.6% (309/8,583)	8% (312/3,918)	14.8% (208/1,408)	**2.3%** (1,023/44,672)

TABLE IV: Proportion of confirmed cases from Table III by age group. This corresponds to the case demographics shown in Fig. 1.

Age	0–9	10–19	20–29	30–39	40–49	50–59	60–69	70–79	$\geq$ 80
Italy	0.5%	1.0%	3.5%	5.6%	10.7%	17.4%	17.7%	**21.4%**	**18.4%**
China	**0.9%**	**1.2%**	**8.1%**	**17.0%**	**19.2%**	**22.4%**	**19.2%**	8.8%	3.2%

TABLE V: Age demographic of the general population for Italy and China.

Age	0–9	10–19	20–29	30–39	40–49	50–59	60–69	70–79	$\geq$ 80
Italy	8.3%	9.5%	10.1%	11.6%	14.9%	**15.8%**	**12.4%**	**10%**	**7.5%**
China	**11.9%**	**11.6%**	**12.9%**	**15.9%**	**15%**	15.4%	10.5%	5%	1.8%

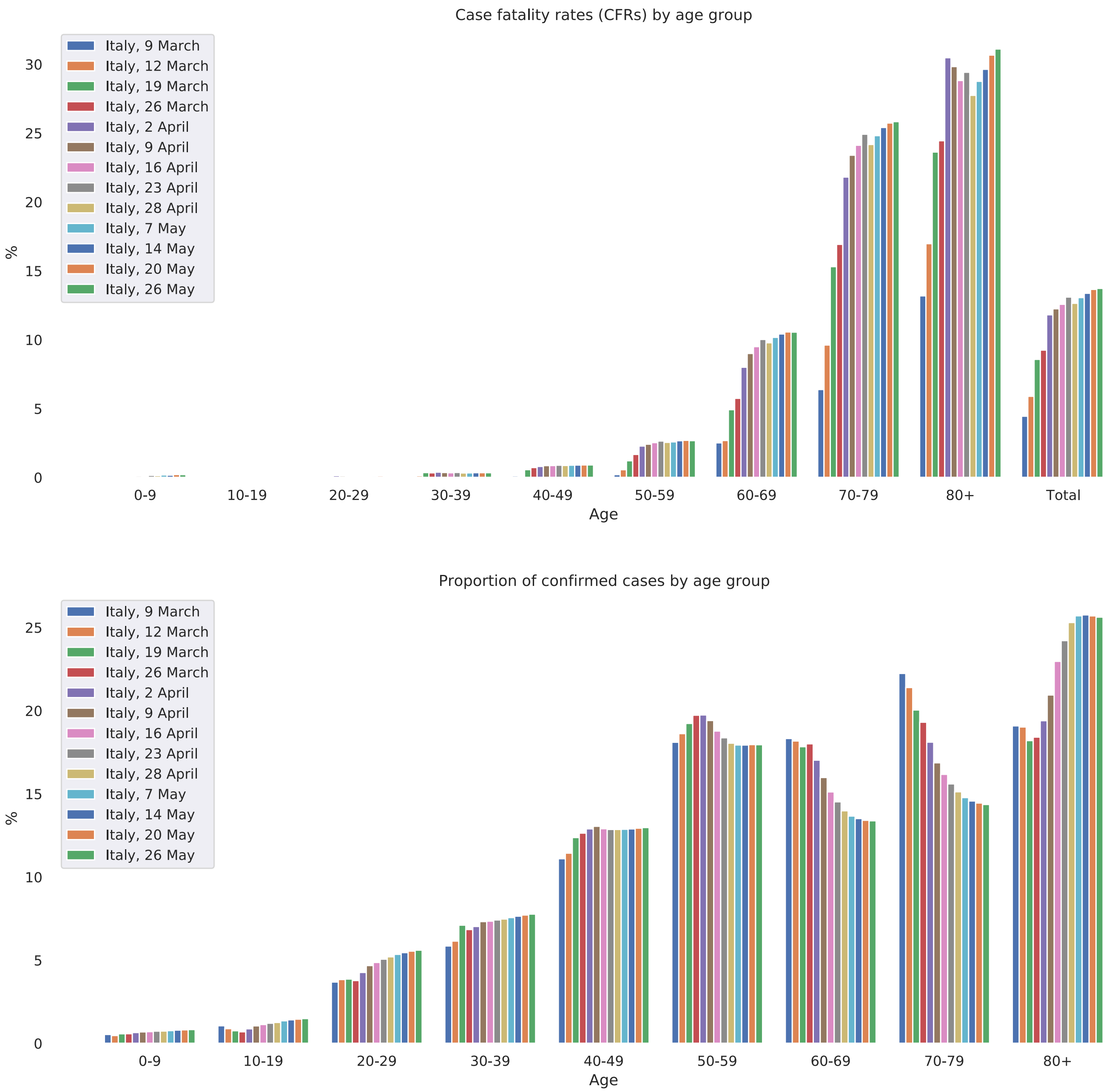

Fig. 8: Different snapshots from Italy show the temporal evolution of CFRs by age group (top) and case demographic (bottom) over the time period for which different causal effects with China as a control country are shown in Fig. 3.

E. Additional results and figures

1) Temporal CFR *data for Spain:* We perform a similar analysis of the temporal evolution of different causal effects of changing country from China to Spain, as done for Italy in §V and Fig. 3. The results are shown in Fig. 9. Recall that the control China remains fixed throughout so that any changes can be attributed to changes in the Spanish data.

Interestingly, a reversal in the sign of the NDE can also be observed for Spain, taking place around 30 March. This bears similarity to the reversal of NDE observed for Italy. The initial increase in NDE is also reflected in the age-specific CFRs shown in the middle of Fig. 9 which are initially increasing for most age groups. Unlike Italy, however, NDE and TCE do not increase monotonically, but reach a maximum (over the time period considered) around 23 April and subsequently decrease again. The NIE also appears less constant than for the case of changing country to Italy shown in Fig. 3, steadily climbing from initially 2.3% to 3.1% at the end of May (ca. 35% increase).

As a remark of caution, we point out that the total number of fatalities reported by the Spanish ministry in age-stratified form is considerably lower than the number of fatalities reported (without separation into age groups) by different sources such as, e.g., [2]. This may have different reasons such as, e.g., latency in their reporting of fatalities in general, or of the exact age group of deceased patients specifically. As a result, CFRs from Spain are lower than other sources suggest, and may thus not be very reliable.

2) Comparison of age-specific CFR*s and case demographic between different countries:* A visual comparison of CFRs by age group and case demographic (similar to that shown in Fig. 1 for only China and Italy) for all different countries in our dataset is shown in Fig. 10.

3) TCEs between different countries: In addition to the pair-wise NDEs and NIEs between the different countries in our dataset, we also show the pair-wise TCEs for completeness in Fig. 11. Note that—as opposed to NDE and NIE—the TCE is, by definition, symmetric, i.e., $\text{TCE}_{0\rightarrow1} = -\text{TCE}_{1\rightarrow0}$, as can be seen from Fig. 11.

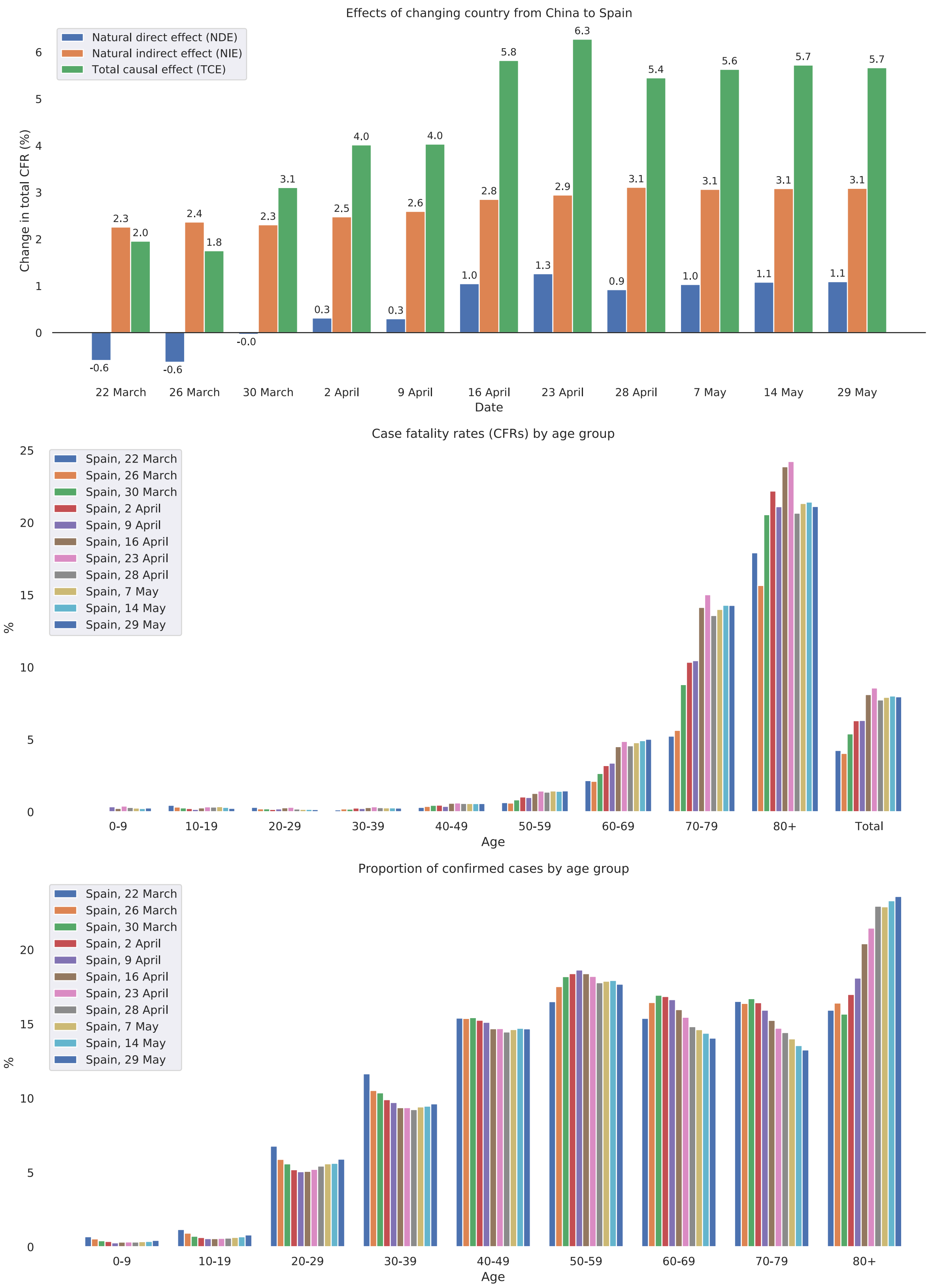

Fig. 9: (top) We use different snapshots from Spain to trace TCE, NDE, and NIE of changing country from China to Spain over a time period of 9 weeks, similar to what is shown in Fig. 3 for Italy. We also show the underlying evolution of CFRs by age group (middle) and case demographic (bottom) for the time points considered.

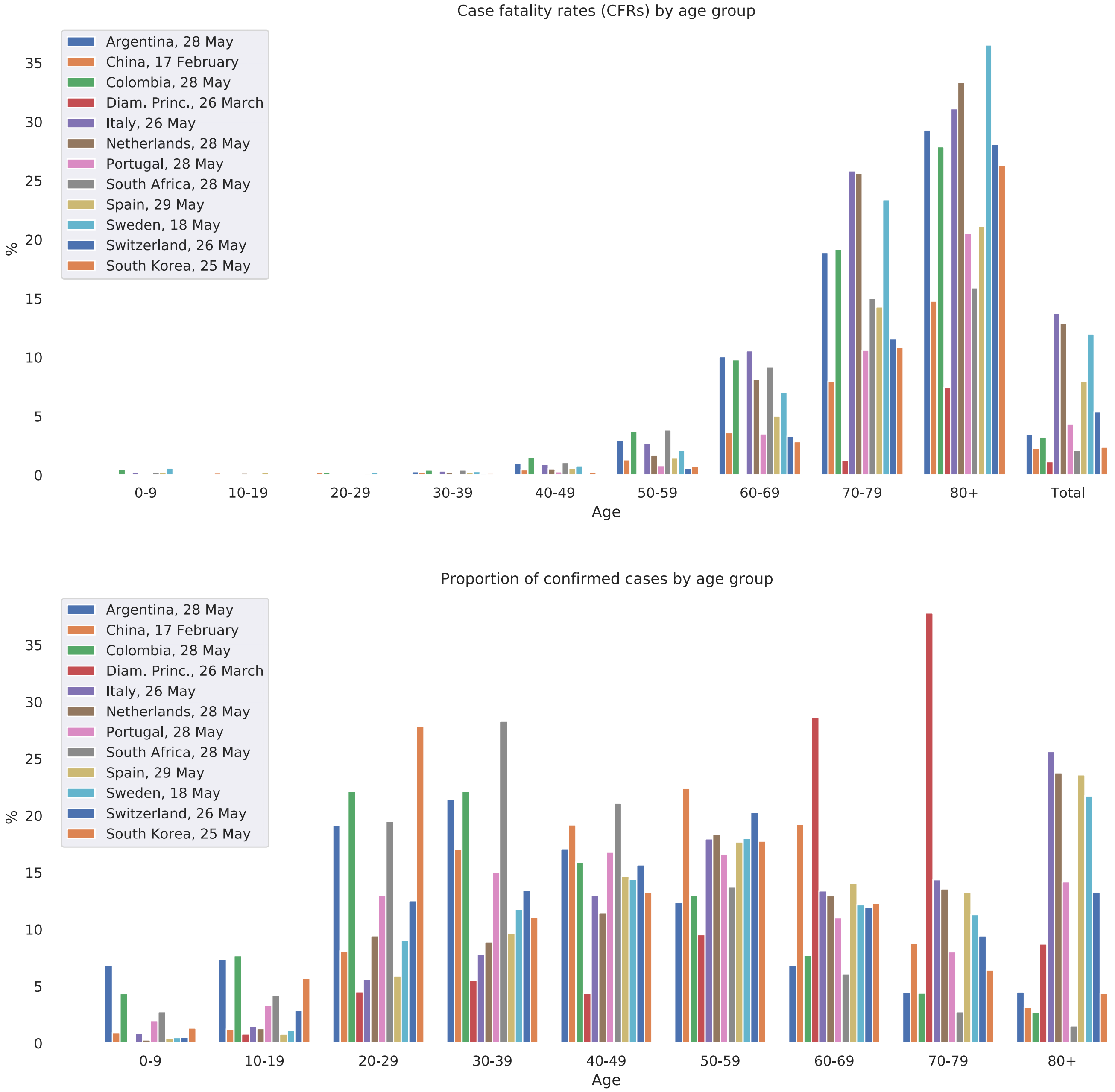

Fig. 10: Comparison of CFRs by age group (top) and case demographic (bottom) for all different countries included in our dataset.

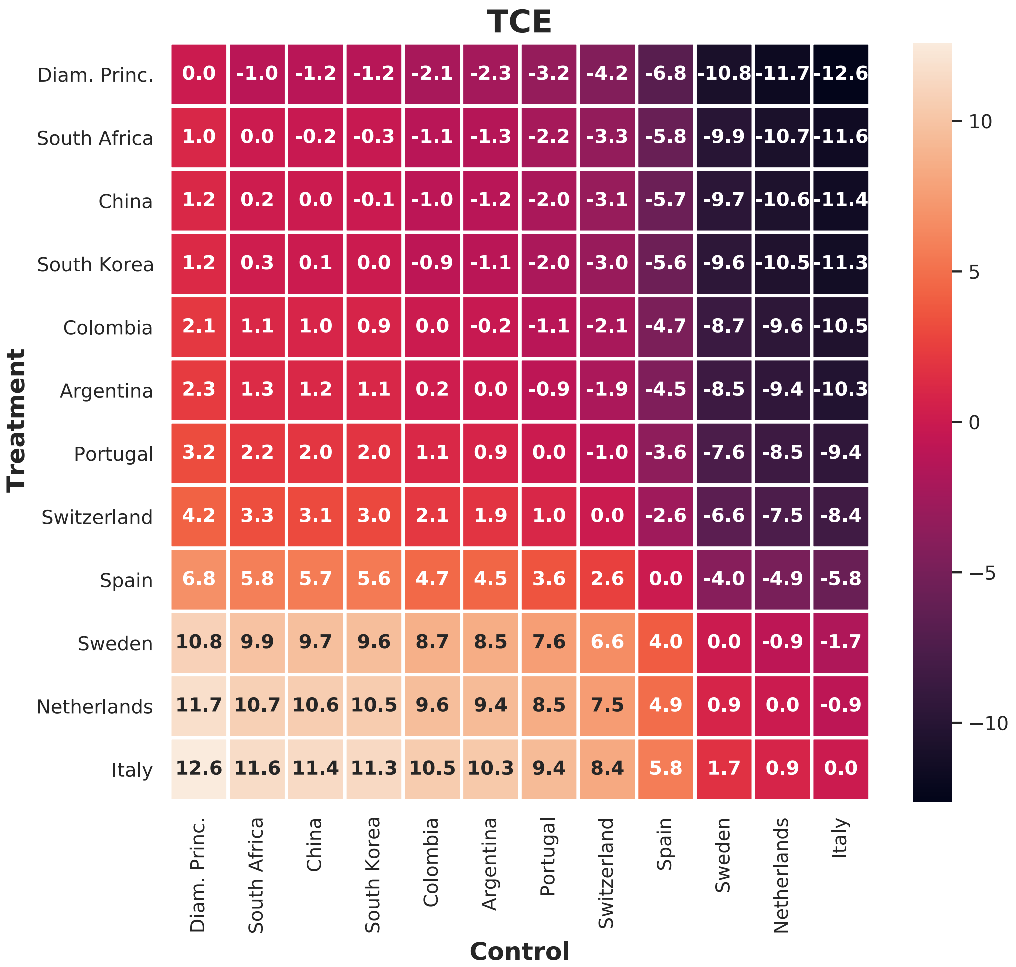

Fig. 11: Total causal effects (TCEs) for switching from the control country (columns) to the treatment country (rows). Numbers show the change in total CFR in %, i.e., negative numbers indicate that switching to the treatment country's approach in terms of *both* CFRs by age group *and* case demographic would lead to a decrease in total CFR. Countries are ordered by their average treatment effect over the remaining 11 data points as a control.

F. Dataset details

In this Appendix, we provide further details on the datasets of age-stratified case and fatality numbers curated as part of this work. We provide three different datasets:

- A dataset containing the latest age-stratified case and fatality numbers for all different countries considered in our analysis, described in more detail in F1.
- A dataset containing longitudinal age-stratified case and fatality numbers for Italy, described in more detail in F2.
- A dataset containing longitudinal age-stratified case and fatality numbers for Spain, described in more detail in F3.

All datasets are contained in the supplementary material in multiple commonly-used formats (`.csv`, `.xlsx`, `.json`, `.pkl`) and will be made publicly available upon publication.

1) Dataset of latest age-stratified case and fatality numbers for different countries: An overview of the dataset of latest age-stratified case and fatality numbers for different countries in the form of metadata is shown in Table VI.

CFRs and absolute case and fatality numbers in age-stratified form are shown in Table VII.

Case demographics are shown in Table VIII.

TABLE VI: Information on the sources for the data regarding the countries in the case study.

Country	Date of reporting	Confirmed cases	Fatalities	Source
Argentina	28 May	14,675	507	[66]
China	17 February	44,672	1023	[4]
Colombia	28 May	25,366	822	[67]
Diam. Princ.	26 March	619	7	[27]
Italy	26 May	230,760	31676	[68]
Netherlands	28 May	45,947	5903	[69]
Portugal	28 May	31,596	1369	[70]
South Africa	28 May	27,280	577	[71]
South Korea	25 May	11,190	266	[72]
Spain	29 May	258,760	20585	[73]
Sweden	18 May	34,432	4125	[74]
Switzerland	26 May	30,707	1648	[75]

TABLE VII: Exact numbers for the comparison of case fatality rates (CFRs) by age group for all countries discussed in §V. Absolute numbers of fatalities/confirmed cases are shown in brackets below.

Age	0–9	10–19	20–29	30–39	40–49	50–59	60–69	70–79	≥ 80	Total
Argentina	0.0% (0/1,002)	0.1% (1/1,080)	0.0% (1/2,813)	0.3% (9/3,142)	1.0% (24/2,508)	3.0% (54/1,812)	10.0% (101/1,005)	18.9% (123/651)	29.3% (194/662)	3.5% (507/14,675)
China	0.0% (0/416)	0.2% (1/549)	0.2% (7/3,619)	0.2% (18/7,600)	0.4% (38/8,571)	1.3% (130/10,008)	3.6% (309/8,583)	8.0% (312/3,918)	14.8% (208/1,408)	2.3% (1,023/44,672)
Colombia	0.5% (5/1,105)	0.1% (1/1,950)	0.2% (13/5,614)	0.4% (24/5,615)	1.5% (61/4,033)	3.7% (121/3,286)	9.8% (192/1,961)	19.2% (214/1,117)	27.9% (191/685)	3.2% (822/25,366)
Diam. Princ.	0.0% (0/1)	0.0% (0/5)	0.0% (0/28)	0.0% (0/34)	0.0% (0/27)	0.0% (0/59)	0.0% (0/177)	1.3% (3/234)	7.4% (4/54)	1.1% (7/619)
Italy	0.2% (4/1,919)	0.0% (0/3,442)	0.1% (12/12,933)	0.3% (62/17,934)	0.9% (273/29,942)	2.7% (1,109/41,435)	10.6% (3,259/30,880)	25.8% (8,562/33,141)	31.1% (18,395/59,134)	13.7% (31,676/230,760)
Netherlands	0.0% (0/128)	0.2% (1/587)	0.1% (3/4,336)	0.2% (10/4,093)	0.5% (28/5,269)	1.7% (142/8,437)	8.1% (484/5,949)	25.6% (1,596/6,229)	33.3% (3,639/10,919)	12.8% (5,903/45,947)
Portugal	0.0% (0/626)	0.0% (0/1,052)	0.0% (1/4,114)	0.0% (1/4,736)	0.3% (15/5,315)	0.8% (42/5,253)	3.5% (122/3,484)	10.6% (269/2,537)	20.5% (919/4,479)	4.3% (1,369/31,596)
South Africa	0.3% (2/755)	0.1% (1/1,147)	0.1% (4/5,319)	0.4% (33/7,720)	1.1% (61/5,754)	3.8% (144/3,753)	9.2% (153/1,663)	15.0% (113/754)	15.9% (66/415)	2.1% (577/27,280)
South Korea	0.0% (0/149)	0.0% (0/636)	0.0% (0/3,117)	0.2% (2/1,235)	0.2% (3/1,481)	0.8% (15/1,987)	2.8% (39/1,375)	10.8% (78/719)	26.3% (129/491)	2.4% (266/11,190)
Spain	0.3% (3/1,123)	0.2% (5/2,068)	0.2% (24/15272)	0.3% (65/24,902)	0.6% (218/37,970)	1.4% (663/45750)	5.0% (1,825/36,355)	14.3% (4,896/34,294)	21.1% (12,886/61,026)	8.0% (20,585/258,760)
Sweden	0.6% (1/168)	0.0% (0/401)	0.3% (8/3,104)	0.3% (12/4,051)	0.8% (39/4,962)	2.1% (129/6,190)	7.0% (294/4,186)	23.4% (909/3,888)	36.5% (2,733/7,482)	12.0% (4,125/34,432)
Switzerland	0.0% (0/162)	0.0% (0/877)	0.0% (0/3,844)	0.1% (5/4,136)	0.1% (4/4,809)	0.6% (37/6,232)	3.3% (121/3,671)	11.6% (335/2,896)	28.1% (1,146/4,080)	5.4% (1,648/30,707)

TABLE VIII: Proportion of confirmed cases by age group for all of the countries considered in section V.

Age	0–9	10–19	20–29	30–39	40–49	50–59	60–69	70–79	≥ 80
Argentina	6.8%	7.4%	19.2%	21.4%	17.1%	12.3%	6.8%	4.4%	4.6%
China	0.9%	1.2%	8.1%	17.0%	19.2%	22.4%	19.2%	8.8%	3.2%
Colombia	4.4%	7.7%	22.1%	22.1%	15.9%	13.0%	7.7%	4.4%	2.7%
Diam. Princ.	0.2%	0.8%	4.5%	5.5%	4.4%	9.5%	28.6%	37.8%	8.7%
Italy	0.8%	1.5%	5.6%	7.8%	13.0%	18.0%	13.4%	14.4%	25.5%
Netherlands	0.3%	1.3%	9.4%	8.9%	11.5%	18.4%	12.9%	13.6%	23.7%
Portugal	2.0%	3.3%	13.0%	15.0%	16.8%	16.6%	11.0%	8.0%	14.3%
South Africa	2.8%	4.2%	19.5%	28.3%	21.1%	13.8%	6.1%	2.8%	1.4%
South Korea	1.3%	5.7%	27.9%	11.0%	13.2%	17.8%	12.3%	6.4%	4.4%
Spain	0.4%	0.8%	5.9%	9.6%	14.7%	17.7%	14.0%	13.3%	23.6%
Sweden	0.5%	1.2%	9.0%	11.8%	14.4%	18.0%	12.2%	11.3%	21.7%
Switzerland	0.5%	2.9%	12.5%	13.5%	15.7%	20.3%	12.0%	9.4%	13.2%

2) Dataset of longitudinal age-stratified case and fatality numbers for Italy: An overview of the dataset of longitudinal age-stratified case and fatality numbers for Italy, in the form of metadata, is shown in Table IX. The source for all different time points is the same as that shown in Table VI for Italy, queried at the corresponding dates shown in Table IX. CFRs and absolute case and fatality numbers in age-stratified form are shown in Table X. Case demographics are shown in Table XI.

TABLE IX: Metadata for the longitudinal data from Italy.

Date of reporting	Confirmed cases	Fatalities
9 March	8,026	357
12 March	13,317	785
19 March	35,529	3,047
23 March	57,695	5,018
26 March	73,534	6,801
2 April	106,231	12,548
9 April	135,968	16,653
16 April	159,003	19,994
23 April	177,025	23,118
28 April	199,389	25,215
7 May	214,047	27,955
14 May	222,022	29,691
20 May	227,153	31,017
26 May	230,760	31,676

TABLE X: Age-specific CFRs for the longitudinal data for Italy.

Age	0–9	10–19	20–29	30–39	40–49	50–59	60–69	70–79	$\geq$ 80	Total
9 March	0.0% (0/43)	0.0% (0/85)	0.0% (0/296)	0.0% (0/470)	0.1% (1/891)	0.2% (3/1453)	2.5% (37/1471)	6.4% (114/1785)	13.2% (202/1532)	4.4% (357/8026)
12 March	0.0% (0/63)	0.0% (0/118)	0.0% (0/511)	0.1% (1/819)	0.1% (1/1523)	0.6% (14/2480)	2.7% (65/2421)	9.6% (274/2849)	17.0% (430/2533)	5.9% (785/13317)
19 March	0.0% (0/205)	0.0% (0/270)	0.0% (0/1374)	0.4% (9/2525)	0.6% (25/4396)	1.2% (83/6834)	4.9% (312/6337)	15.3% (1090/7121)	23.6% (1528/6467)	8.6% (3047/35529)
23 March	0.0% (0/318)	0.0% (0/386)	0.0% (0/2192)	0.3% (12/3995)	0.6% (41/7267)	1.5% (168/11280)	5.2% (541/10423)	15.6% (1768/11320)	23.7% (2488/10514)	8.7% (5018/57695)
26 March	0.0% (0/428)	0.0% (0/512)	0.0% (0/2778)	0.3% (17/5033)	0.7% (67/9295)	1.7% (243/14508)	5.7% (761/13243)	16.9% (2403/14198)	24.4% (3310/13539)	9.2% (6801/73534)
2 April	0.0% (0/693)	0.0% (0/931)	0.1% (6/4530)	0.4% (29/7466)	0.8% (110/13701)	2.3% (479/20975)	8.0% (1448/18089)	21.8% (4196/19238)	30.5% (6280/20608)	11.8% (12548/106231)
9 April	0.1% (1/938)	0.0% (0/1432)	0.1% (7/6360)	0.4% (36/9956)	0.9% (153/17745)	2.4% (638/26391)	9.0% (1957/21734)	23.4% (5366/22934)	29.8% (8495/28478)	12.2% (16653/135968)
16 April	0.1% (1/1123)	0.0% (0/1804)	0.1% (7/7737)	0.3% (40/11686)	0.9% (178/20519)	2.5% (756/29858)	9.5% (2284/24040)	24.1% (6203/25717)	28.8% (10525/36519)	12.6% (19994/159003)
23 April	0.2% (2/1304)	0.0% (0/2146)	0.1% (7/8963)	0.4% (48/13137)	0.9% (203/22767)	2.6% (861/32524)	10.0% (2576/25707)	24.9% (6882/27615)	29.4% (12609/42862)	13.1% (23188/177025)
28 April	0.1% (2/1478)	0.0% (0/2511)	0.1% (8/10377)	0.3% (49/14907)	0.9% (224/25644)	2.6% (918/35986)	9.8% (2727/27880)	24.2% (7291/30158)	27.7% (13996/50448)	12.6% (25215/199389)
7 May	0.2% (3/1642)	0.0% (0/2908)	0.1% (9/11457)	0.3% (54/16189)	0.9% (246/27553)	2.6% (993/38399)	10.2% (2976/29252)	24.8% (7849/31627)	28.8% (15825/55020)	13.1% (27955/214047)
14 May	0.2% (3/1774)	0.0% (0/3148)	0.1% (12/12115)	0.3% (59/16981)	0.9% (258/28627)	2.7% (1063/39822)	10.4% (3127/30010)	25.4% (8221/32353)	29.6% (16948/57192)	13.4% (29691/222022)
20 May	0.2% (4/1851)	0.0% (0/3312)	0.1% (14/12599)	0.3% (61/17528)	0.9% (268/29390)	2.7% (1101/40803)	10.6% (3219/30466)	25.7% (8447/32824)	30.7% (17903/58380)	13.7% (31017/227153)
26 May	0.2% (4/1919)	0.0% (0/3442)	0.1% (12/12933)	0.3% (62/17934)	0.9% (273/29942)	2.7% (1109/41435)	10.6% (3259/30880)	25.8% (8562/33141)	31.1% (18395/59134)	13.7% (31676/230760)

TABLE XI: Proportion of confirmed cases by age group for longitudinal data for Italy.

Age	0–9	10–19	20–29	30–39	40–49	50–59	60–69	70–79	$\geq$ 80
9 March	0.5%	1.1%	3.7%	5.9%	11.1%	18.1%	18.3%	22.2%	19.1%
12 March	0.5%	0.9%	3.8%	6.2%	11.4%	18.6%	18.2%	21.4%	19.0%
19 March	0.6%	0.8%	3.9%	7.1%	12.4%	19.2%	17.8%	20.0%	18.2%
23 March	0.6%	0.7%	3.8%	6.9%	12.6%	19.6%	18.1%	19.6%	18.1%
26 March	0.6%	0.7%	3.8%	6.8%	12.6%	19.7%	18.0%	19.3%	18.5%
2 April	0.7%	0.9%	4.3%	7.0%	12.9%	19.7%	17.0%	18.1%	19.4%
9 April	0.7%	1.1%	4.7%	7.3%	13.1%	19.4%	16.0%	16.9%	20.8%
16 April	0.7%	1.1%	4.9%	7.3%	12.9%	18.8%	15.1%	16.2%	23.0%
23 April	0.7%	1.2%	5.1%	7.4%	12.9%	18.4%	14.5%	15.6%	24.2%
28 April	0.7%	1.3%	5.2%	7.5%	12.9%	18.0%	14.0%	15.1%	25.3%
7 May	0.8%	1.4%	5.4%	7.6%	12.9%	17.9%	13.7%	14.7%	25.6%
14 May	0.8%	1.4%	5.5%	7.6%	12.9%	17.9%	13.5%	14.6%	25.8%
20 May	0.8%	1.5%	5.5%	7.7%	12.9%	18.0%	13.4%	14.5%	25.7%
26 May	0.8%	1.5%	5.6%	7.8%	13.0%	18.0%	13.4%	14.4%	25.5%

3) Dataset of longitudinal age-stratified case and fatality numbers for Spain: An overview of the dataset of longitudinal age-stratified case and fatality numbers for Spain, in the form of metadata, is shown in Table XII. The source for all different time points is the same as that shown in Table VI for Spain, queried at the corresponding dates shown in Table XII.

CFRs and absolute case and fatality numbers in age-stratified form are shown in Table XIII.

Case demographics are shown in Table XIV.

TABLE XII: Metadata for the longitudinal data from Spain.

Date of reporting	Confirmed cases	Fatalities
22 March	18,959	805
26 March	32,816	1,326
30 March	51,626	2,784
2 April	69,177	4,361
9 April	106,447	6,729
16 April	133,082	10,793
23 April	152,687	13,078
28 April	204,866	15,853
7 May	220,444	17,460
14 May	239,095	19,115
29 May	258,760	20,585

TABLE XIII: Longitudinal age-stratified data for Spain.

Age	0–9	10–19	20–29	30–39	40–49	50–59	60–69	70–79	≥ 80	Total
22 March	0.0% (0/129)	0.5% (1/221)	0.3% (4/1285)	0.1% (3/2208)	0.3% (9/2919)	0.6% (20/3129)	2.2% (63/2916)	5.2% (164/3132)	17.9% (541/3020)	4.2% (805/18959)
26 March	0.0% (0/175)	0.3% (1/302)	0.2% (4/1932)	0.2% (7/3454)	0.4% (19/5045)	0.6% (35/5749)	2.1% (114/5397)	5.6% (303/5377)	15.7% (843/5385)	4.0% (1326/32816)
30 March	0.0% (0/212)	0.3% (1/368)	0.2% (6/2883)	0.2% (10/5351)	0.5% (36/7965)	0.8% (78/9390)	2.7% (232/8744)	8.8% (759/8625)	20.5% (1662/8088)	5.4% (2784/51626)
2 April	0.0% (0/250)	0.2% (1/434)	0.2% (6/3590)	0.3% (18/6853)	0.5% (49/10551)	1.0% (131/12722)	3.2% (373/11657)	10.3% (1176/11368)	22.2% (2607/11752)	6.3% (4361/69177)
9 April	0.4% (1/285)	0.2% (1/588)	0.2% (11/5381)	0.2% (24/10341)	0.4% (61/16088)	1.0% (197/19836)	3.4% (597/17713)	10.5% (1773/16957)	21.1% (4064/19258)	6.3% (6729/106447)
16 April	0.2% (1/423)	0.3% (2/734)	0.3% (19/6763)	0.3% (37/12466)	0.6% (116/19536)	1.3% (312/24471)	4.5% (958/21249)	14.1% (2868/20287)	23.9% (6480/27153)	8.1% (10793/133082)
23 April	0.4% (2/502)	0.3% (3/869)	0.3% (25/7962)	0.3% (50/14304)	0.6% (138/22430)	1.4% (400/27795)	4.9% (1149/23595)	15.0% (3374/22470)	24.2% (7937/32760)	8.6% (13078/152687)
28 April	0.3% (2/660)	0.3% (4/1206)	0.2% (22/11138)	0.3% (55/18924)	0.6% (172/29629)	1.4% (497/36423)	4.6% (1387/30361)	13.6% (4012/29550)	20.7% (9702/46975)	7.7% (15853/204866)
7 May	0.3% (2/765)	0.4% (5/1398)	0.2% (21/12321)	0.3% (57/20759)	0.6% (185/32239)	1.4% (569/39418)	4.8% (1541/32226)	14.0% (4320/30861)	21.3% (10760/50457)	7.9% (17460/220444)
14 May	0.2% (2/871)	0.3% (5/1619)	0.2% (23/13439)	0.3% (62/22643)	0.6% (201/35175)	1.4% (610/42874)	4.9% (1693/34380)	14.3% (4628/32395)	21.4% (11931/55699)	8.0% (19155/239095)
29 May	0.3% (3/1123)	0.2% (5/2068)	0.2% (24/15272)	0.3% (65/24902)	0.6% (218/37970)	1.4% (663/45750)	5.0% (1825/36355)	14.3% (4896/34294)	21.1% (12886/61026)	8.0% (20585/258760)

TABLE XIV: Proportion of confirmed cases by age group for the longitudinal data from Spain.

Age	0–9	10–19	20–29	30–39	40–49	50–59	60–69	70–79	$\geq$ 80
22 March	0.5%	2.9%	12.5%	13.5%	15.7%	20.3%	12.0%	9.4%	13.2%
26 March	0.5%	0.9%	5.9%	10.5%	15.4%	17.5%	16.4%	16.4%	16.5%
30 March	0.4%	0.7%	5.6%	10.4%	15.4%	18.2%	16.9%	16.7%	15.7%
2 April	0.4%	0.6%	5.2%	9.9%	15.3%	18.4%	16.9%	16.4%	16.9%
9 April	0.3%	0.6%	5.1%	9.7%	15.1%	18.6%	16.6%	15.9%	18.1%
16 April	0.3%	0.6%	5.1%	9.4%	14.7%	18.4%	16.0%	15.2%	20.3%
23 April	0.3%	0.6%	5.2%	9.4%	14.7%	18.2%	15.5%	14.7%	21.4%
28 April	0.3%	0.6%	5.4%	9.2%	14.5%	17.8%	14.8%	14.4%	23.0%
7 May	0.3%	0.6%	5.6%	9.4%	14.6%	17.9%	14.6%	14.0%	23.0%
14 May	0.4%	0.7%	5.6%	9.5%	14.7%	17.9%	14.4%	13.5%	23.3%
29 May	0.4%	0.8%	5.9%	9.6%	14.7%	17.7%	14.0%	13.3%	23.6%
