# Supplementary material for: Simpson's Paradox in COVID-19 Case Fatality Rates: A Mediation Analysis of Age-Related Causal Effects
Source: IEEE Trans Artif Intell. 2021 Apr 14;2(1):18–27. doi: 10.1109/TAI.2021.3073088 (PMC8791436; doi:10.1109/TAI.2021.3073088)
Supplement: AGE-STRATIFIED COVID-19 CASE FATALITY RATES (CFRS): DIFFERENT COUNTRIES AND LONGITUDINAL [file tai-3073088-mm.zip › tai-3073088-mm/README.pdf]

DESCRIPTION: appendix.pdf
DESCRIPTION: A separate pdf version of the Appendix; this is already contained at the end of the submitted manuscript (i.e., the main pdf file) and coincides with the submitted source files.
data_and_code.zip
DESCRIPTION: A zip archive containing (i) a folder with all datasets introduced in the paper in multiple common formats; and (ii) a Jupyter notebook which can be executed to reproduce all results and figures. (Both of these were already uploaded to the IEEE cloud previously, as indicated during the submission; we only include it here again for completeness.)

SIZE: 3.03 MB

PLAYER INFORMATION: PDF, excel files and codes

PACKING LIST: tai-3073088-mm.zip

CONTACT INFORMATION:

Julius vonKugelgen
Max Planck Institute for Intelligent Systems, Tubingen
Empirical Inference
Tübingen, Germany
Email：julius.von.kuegelgen@tuebingen.mpg.de
